# Protective effects of Gα_i3_ deficiency in a murine heart-failure model of β_1_-adrenoceptor overexpression

**DOI:** 10.1007/s00210-023-02751-8

**Published:** 2023-10-16

**Authors:** Tobias Schröper, Dennis Mehrkens, Veronika Leiss, Frederik Tellkamp, Stefan Engelhardt, Stefan Herzig, Lutz Birnbaumer, Bernd Nürnberg, Jan Matthes

**Affiliations:** 1 Center of Pharmacology, Department II, University of Cologne and University Hospital Cologne, Cologne, Germany; 2 Department of Internal Medicine III, University Hospital of Cologne, Cologne, Germany and Centre for Molecular Medicine Cologne, University of Cologne, Cologne, Germany; 3 Centre for Molecular Medicine Cologne, CMMC, University of Cologne, Cologne, Germany; 4 Department of Pharmacology, Experimental Therapy and Toxicology, Institute for Experimental and Clinical Pharmacology and Pharmacogenomics, and Interfaculty Centre for Pharmacogenomics and Drug Research, Eberhard Karls Universität, Tübingen, Germany; 5 CECAD Research Centre Institute for Genetics, University of Cologne, Cologne, Germany; 6 Institute of Pharmacology and Toxicology, Technische Universität München, Munich, Germany; 7 TH Köln-University of Applied Sciences, Cologne, Germany; 8 Laboratory of Signal Transduction, National Institute of Environmental Health Sciences, Research Triangle Park, Durham, North Carolina USA; 9 Institute of Biomedical Research, School of Medical Sciences, Catholic University of Buenos Aires, Buenos Aires, Argentina

**Keywords:** Adrenergic receptor, G_i_ protein, Cardiomyopathy, Heart failure, Cardioprotection

## Abstract

We have shown that in murine cardiomyopathy caused by overexpression of the β_1_-adrenoceptor, Gα_i2_-deficiency is detrimental. Given the growing evidence for isoform-specific Gα_i_-functions, we now examined the consequences of Gα_i3_ deficiency in the same heart-failure model. Mice overexpressing cardiac β_1_-adrenoceptors with (β_1_-tg) or without Gα_i3_-expression (β_1_-tg/Gα_i3_^−/−^) were compared to C57BL/6 wildtypes and global Gα_i3_-knockouts (Gα_i3_^−/−^). The life span of β_1_-tg mice was significantly shortened but improved when Gα_i3_ was lacking (95% CI: 592–655 vs. 644–747 days). At 300 days of age, left-ventricular function and survival rate were similar in all groups. At 550 days of age, β_1_-tg but not β_1_-tg/Gα_i3_^−/−^ mice displayed impaired ejection fraction (35 ± 18% vs. 52 ± 16%) compared to wildtype (59 ± 4%) and Gα_i3_^−/−^ mice (60 ± 5%). Diastolic dysfunction of β_1_-tg mice was prevented by Gα_i3_ deficiency, too. The increase of ANP mRNA levels and ventricular fibrosis observed in β_1_-tg hearts was significantly attenuated in β_1_-tg/Gα_i3_^−/−^ mice. Transcript levels of phospholamban, ryanodine receptor 2, and cardiac troponin I were similar in all groups. However, Western blots and phospho-proteomic analyses showed that in β_1_-tg, but not β_1_-tg/Gα_i3_^−/−^ ventricles, phospholamban protein was reduced while its phosphorylation increased. Here, we show that in mice overexpressing the cardiac β_1_-adrenoceptor, Gα_i3_ deficiency slows or even prevents cardiomyopathy and increases shortened life span. Previously, we found Gα_i2_ deficiency to aggravate cardiac dysfunction and mortality in the same heart-failure model. Our findings indicate isoform-specific interventions into G_i_-dependent signaling to be promising cardio-protective strategies.

**Supplementary information:**

The online version contains supplementary material available at 10.1007/s00210-023-02751-8.

## Introduction

Heart failure is a major cause of cardiovascular diseases affecting at least 26 million people worldwide (Savarese and Lund 2017). β-Adrenoceptor antagonists are a cornerstone in the therapy of chronic heart failure because some have been proven to reduce mortality independent of age and gender of the patients (Kotecha et al. 2016, 2017). Guarding the heart from (excessive) β-adrenergic stimulation seems to be cardio-protective mainly by preventing G_s_-protein mediated signalling (Baker 2014). G_s_-proteins are the cognate interaction partners of β_1_-adrenoceptors (Xiao et al. 1999; Seyedabadi et al. 2019). Overexpression of β_1_-adrenoceptors (β_1_-AR) in murine hearts has been shown to cause dilative cardiomyopathy leading to severe heart failure (Engelhardt et al. 1999, 2001a). Although cardiac overexpression of β_2_-adrenoceptors (β_2_-AR) also leads to cardiac failure, a significantly higher level of overexpression is required (Liggett et al. 2000). β_2_-Adrenoceptors couple to both G_s_ and G_i_ proteins (Xiao et al. 1999). G_i_ proteins are thought to be involved in the protection against excessive β-adrenergic stimulation in heart failure (Brown and Harding 1992; El-Armouche et al. 2003), and G_i_-protein-mediated signaling downstream from β_2_-AR has been shown to be anti-apoptotic (Chesley et al. 2000). Despite the differences between β-adrenoceptor isoforms regarding G-protein coupling, it should be considered that G_i_ proteins seem to modulate both β_1_- and β_2_-adrenergic signalling (Li et al. 2004; Martin et al. 2004; Melsom et al. 2014). It has to be mentioned that not all studies support the idea of G_i_ proteins mediating the cardio-protective effects of β_2_-adrenoceptor stimulation (Xiao et al. 2003; Ahmet et al. 2005) or of G_i_-protein signaling being cardio-protective in general (Hussain et al. 2013). At least two Gα_i_-isoforms, Gα_i2_ and Gα_i3_, are expressed in the cardiovascular system, which have been shown to interplay (Thompson et al. 2007) and to exhibit redundant but also distinct functions (Gohla et al. 2007; Dizayee et al. 2011; Plummer et al. 2012; Wiege et al. 2012, 2013; Köhler et al. 2014; Wang et al. 2014; Devanathan et al. 2015; Mauriac et al. 2017; Beer-Hammer et al. 2018). Of particular interest, in a murine ischemia–reperfusion model, Köhler et al. showed lack of Gα_i2_ to worsen cardiac damage while lack of Gα_i3_ was beneficial (Köhler et al. 2014). Thus, the increased Gα_i2_-expression observed in failing myocardium might be interpreted as compensatory, while the role of Gα_i3_ remains unclear (Eschenhagen et al. 1992b; Kompa et al. 1999).

In a previous study, we reported that lack of Gα_i2_ (Gα_i2_^−/−^) had detrimental effects in β_1_-transgenic (β_1_-tg) mice (Keller et al. 2015): survival of β_1_-tg/Gα_i2_^−/−^ mice was drastically shortened, and these animals showed a significantly impaired cardiac function. This occurred already at an age of about 300 days, i.e., when β_1_-tg or Gα_i2_^−/−^ mice were unaffected in this regard. Considering the unknown consequences of functional isoform redundancy between the closely related Gα_i2_ and Gα_i3_ proteins on the one hand and isoform-specific, distinct functions on the other hand, we now examined the impact of Gα_i3_ deficiency on cardiac function of β_1_-tg mice. In particular, we asked whether the lack of Gα_i3_ impairs heart function of β_1_-tg mice, is not detrimental, or may even rescue from β_1_-AR-induced cardiomyopathy.

We find Gα_i3_ deficiency to be cardio-protective in terms of slowing down or even preventing the development of β_1_-AR-induced cardiomyopathy. Together with previous findings, our study indicates isoform-specific targeting of Gα_i_-protein-mediated signaling to be a promising novel strategy to treat cardiovascular diseases. Parts of the data have already been published as a conference abstract (Schröper et al. 2020).

## Methods

### Mouse models

Mice with cardiac overexpression of the human β_1_-AR (β_1_-tg) have been described earlier (Engelhardt et al. 1999). We had backcrossed these FVB/N-based transgenic mice to a C57BL/6 J background (Keller et al. 2015). In the current study, β_1_-tg mice were crossbred with mice globally lacking Gα_i3_ (Gohla et al. 2007), to produce β_1_-tg Gα_i3_-deficient mice (β_1_-tg/Gα_i3_^−/−^). Age-matched wildtype and Gα_i3_-deficient (Gα_i3_^−/−^) littermates served as controls. Animals of both sexes were used for our study (sex distribution given in table S1). We kept mice in individually ventilated cages with a 12 h/12 h dark/light cycle and food and water ad libitum. For genotyping, tail or ear clips from 3-week-old mice were processed. Genomic DNA was prepared and genotyping PCR for Gα_i3_ and the β_1_-AR was performed as described previously (Dizayee et al. 2011; Keller et al. 2015). Animals were killed by cervical dislocation. Since, in a previous study, cardiac β_1_-AR overexpression on a C57BL/6 J background by itself had no effect on cardiac function or survival at the age of 300 days (Keller et al. 2015), we chose a second target age to address putative effects of Gα_i3_ deficiency in β_1_-tg mice. Based on our own data and the report of another group, we thus additionally analyzed animals at the age of 550 days (Lee et al. 2015; Keller et al. 2015). The responsible federal state authority approved animal breeding, maintenance and experiments (Landesamt fuer Natur-, Umwelt- und Verbraucherschutz Nordrhein-Westfalen; references: 84–02.05.20.12.294, 84–02.05.20.13.060, and 84–02.04.2016.A422). All animal experiments complied with the guidelines from Directive 2010/63/EU of the European Parliament on the protection of animals used for scientific purposes.

### Ventricle-to-body-weight ratio

Non-fasting mice were weighed directly before being killed. Immediately after cervical dislocation, we removed the heart, cut the atria and eliminated remaining intraventricular blood. We analyzed mice at an age of 304 ± 7 days and at the second target age of 553 ± 6 days, including mice just examined by echocardiography.

### Histology and histomorphometrical analysis of fibrotic area

Only mice at the advanced age (553 ± 3 days) were used for this analysis. Cryo-Sects (6 μm thickness) were obtained from excised hearts frozen in liquid nitrogen, fixed in ice-cold acetone, subsequently immersed in Roti®-Histol for 10 min at room temperature, and transferred to water through descending concentrations of ethanol (100%, 96%, 75%). Staining was performed using a 0.1% solution of Sirius Red F3BA in saturated aqueous solution of picric acid for 45 min at 25 °C. Subsequently, slices were rinsed in 1% acetic acid for 2 min. Sections were dehydrated in ascending concentrations of ethanol (75%, 96%, and 100%, each 1 min) and cleared in two stages in Roti®-Histol, 10 min each. Sections were covered with Roti®-Histokitt mounting medium (Carl Roth, Karlsruhe, Germany) and a glass cover slip. After scanning the Picro Sirius Red sections with the Keyence BZ-9000E microscope, images were taken at mid-ventricular level (× 20 magnification), and interstitial fibrosis was quantified as percentage of total tissue area in the field of view. Planimetry was performed using a Keyence BZ2-Analyser software using hybrid cell count algorithm (Keyence, Osaka, Japan).

### Echocardiography

Echocardiography was performed using the high-frequency VisualSonics Vevo® 3100 Imaging System (Fujifilm) with a MX550D transducer (22–55 MHz; axial resolution: 40 µm). Mice were prepared and examined under light inhalation anesthesia with oxygen and 1.5% isoflurane through a nose cap. Chest and upper abdominal hair was shaved, and the mice were placed on a warmed platform to maintain physiological conditions. We monitored ECG, heart rate, core temperature, and respiratory frequency. Systolic parameters were obtained by using the B- and M-Mode in parasternal long and short axis views of the left ventricle. Doppler flow profiles were acquired to estimate the isovolumic relaxation time (IVRT), an indicator of diastolic ventricular function. We evaluated the ultrasound imaging data by working with the software Vevo LAB (Fujifilm). Strain analyses via Speckle Tracking were performed by using the Vevo Strain Software (Fujifilm). Younger mice used for echocardiographic investigation were 303 ± 4, older 551 ± 13 days of age.

### Survival analysis

Survival was analyzed by Kaplan–Meier estimation and log-rank test. We defined a priori “spontaneous” death as the event of interest, while being killed for any reason (e.g., organ removal) and survival at the end of the study were considered censored events. Mice used for breading were not included into the survival analysis. Total numbers of mice included in our analysis were 408, 262, 157, and 82 for wildtype, β_1_-tg, Gα_i3_^−/−^, and β_1_-tg/Gα_i3_^−/−^, respectively. Numbers of events of interest during the period of observation were 17, 52, 11, and 13, respectively.

### Quantitative real-time PCR

Quantitative real-time PCR (qPCR) was used to reveal the relative ventricular mRNA-expression levels of the G_i_ isoforms Gα_i2_ (*Gnai2*) and Gα_i3_ (*Gnai3*), the cardiomyopathy markers atrial natriuretic peptide ANP (*Nppa*) and brain natriuretic peptide BNP (*Nppb*), and the phosphorylation targets of protein kinase A (PKA) ryanodine receptor 2 (*Ryr2*), phospholamban (*Pln*), and troponin I (*Tnni3*). Ventricles were stored at − 80 °C until mRNA-isolation. All procedures were performed according to the manufacturer’s protocol (QIAGEN, Hilden, Germany). The RNeasy® Fibrous Tissue Kit (QIAGEN) was used to isolate the mRNA. Quality and quantity of the purified mRNA were controlled by NanoDrop 8000 Spectrophotometer (Thermo Scientific, Waltham, MA, USA). Reverse transcription was done by using the QuantiTect® Reverse Transcription Kit (QIAGEN). All qPCRs were run in triplicates with the ORA™ qPCR Green ROX L Mix, 2X Kit (highQu). Primer pairs for *Gnai2*, *Gnai3*, *Nppa*, *Nppb*, *Ryr2*, *Pln*, and *Tnni3* have been reported before (Dizayee et al. 2011; Wiege et al. 2012; Bai et al. 2013; Keller et al. 2015) and are listed in Table S2. The gene encoding 40S ribosomal protein S29 (*Rps29*) served as a housekeeping gene (Figure S1). The qPCR was initiated with incubation at 95 °C for 15 min. Next, 45 cycles of denaturation were conducted at 95 °C for 15 s. Subsequently, annealing at 60 °C for 25 s, and elongation at 72 °C for 10 s were applied with a transition rate of 20 °C per second. A melting curve analysis was performed at the end to control the product purity at 64 °C for 1 min with a transition rate of 0.1 °C per second. Younger mice used for mRNA-analyses were 301 ± 4, older 554 ± 5 days of age.

### Western blot analysis

Liquid-frozen ventricles were homogenized in 500 μl protein lysis buffer (20 mmol/l Tris, pH 8.3; 0.67% SDS; 238 mmol/l 2-mercaptoethanol; 0.2 mmol/l PMSF). Electrophoretic separation of Gα_i_ isoforms was performed in gels containing 6 M urea (Gohla et al. 2007). The proteins were visualized by immunodetection using the following primary antibodies described elsewhere (Beer-Hammer et al. 2018): rabbit anti-Gα_i1/i2_ (7.2 ng/ml) (Leiss et al. 2020), rabbit anti-Gα_i3_ (50 ng/ml) (Vega et al. 2020). The protein levels of Gα_i2_ and Gα_i3_ were quantified using densitometric analysis software (Image Lab; Bio-Rad, Gräfelfing, Germany) and were normalized to the levels of GAPDH (#2118; Cell Signalling Technology, Frankfurt, Germany) of the same samples. Twenty micrograms protein per lane were loaded. The membranes were first stained with the Gα_i2_ or Gα_i3_ antibody, respectively. Membranes were than stripped and stained with the Akt antibody, stripped again, and subsequently stained with the GAPDH antibody to control for equal loading. Ventricles from three animals per genotype were analyzed in three independent experiments. For size orientation, protein standards were loaded (BioRad Precision Plus Protein Standard Dual Colour, and Nippon Genetics BlueStar PLUS Prestained Protein Standard). For the analysis of Akt phosphorylation, we used rabbit antibodies recognizing either total Akt or pAkt only when phosphorylated at Ser473 (#9272 and #9271; Cell Signalling Technology). Phospholamban expression was determined using a mouse monoclonal antibody provided by Badrilla Ltd. (#A010-14). Younger mice used for Western blot analyses were 307 ± 8, older mice 552 ± 8 days of age.

### Myocyte preparation for proteomics analyses

We isolated ventricular myocytes from wildtype, β_1_-tg, Gα_i3_^−/−^, and β_1_-tg/Gα_i3_^−/−^ mice (*n* = 3 each; age: 200 ± 56 days). Isolation followed a modified procedure according to Ackers-Johnson et al. (Ackers-Johnson et al. 2016). The chest of anesthetized mice was opened to expose the heart. Descending aorta was cut, and the heart was immediately flushed by injection of 7 ml EDTA buffer into the right ventricle. The heart was removed, and ascending aorta was retrogradely cannulated. Digestion was achieved by sequential injection of 10 ml EDTA buffer, 3 ml perfusion buffer, and 20 ml Liberase buffer (Roche, Liberase TM 0.05 mg/ml) via coronary circulation. Ventricles were then gently pulled into 1-mm pieces using forceps. Cellular dissociation was completed by gentle trituration, followed by addition of 5 ml stop buffer (perfusion buffer containing 5% FBS). Cell suspension was passed through a 100-μm filter, and cells underwent 4 sequential rounds of gravity settling, using perfusion buffer. The supernatant was discarded. The cell pellet in each round was enriched with myocytes and ultimately formed a highly pure myocyte fraction. Cardiomyocyte yields and percentage of viable rod-shaped cells were controlled under an inverse microscope. The final pellet was lysed in buffer (4% SDS in 100 mM Tris/HCl, pH 7.6). Lysates were homogenized, heated at 70 °C for 10 min, and clarified by centrifugation and protein concentrations were determined using the Bio-Rad DC assay. Proteins (1 mg) were precipitated with acetone for 1 h at – 20 °C. The pellet was washed with 80% acetone once and resuspended in 8 M urea buffer (6 M thiourea, 2 M urea in 10 mM HEPES pH 7.5). Proteins were reduced with DTT (10 mM), alkylated with IAA (55 mM) and digested for 3 h with LysC (1:50 enzyme:substrate ratio, Wako chemicals). For further digestion with Trypsin (1:100 enzyme:substrate ratio, Promega), samples were diluted with ammonium bicarbonate buffer (50 mM). For whole proteomics analysis, aliquots of 50 µg were taken and desalted on stage tips. For phospho-proteomic analysis, the remaining 950 µg peptide solution was desalted using SepPak C18 cartridges and dried, and phospho-peptides were enriched using the High-Select TiO2 Phosphopeptide Enrichment Kit (Thermo scientific) following the manufacturer’s instruction.

### Proteomics analyses: sample measurement and data processing

Samples were measured on a Q Exactive Plus Hybrid Quadrupol-Orbitrap mass spectrometer (MS) coupled to an EASY-nLC 1000 UHPLC (Thermo Fisher Scientific) and analyzed with a 240 min gradient using Top 10 DDA method. Phospho-proteomic samples were analyzed with a 90 min gradient using Top 10 DDA method. Raw MS data files were analyzed using MaxQuant software (Max Planck Institute of Biochemistry, Martinsried, Germany) (Cox and Mann 2008). We used the Uniprot Mouse database (release November 2019) extended by the human β_1_-AR (hADRB1) sequence for spectral matching. Default settings were used, and peptides and proteins were identified using a false discovery rate (FDR) of 1%. As variable modification, p(STY) was enabled. To appreciate the biological significance of the differentially phosphorylated proteins, the ingenuity pathway analysis (IPA, QIAGEN, Germany) was used to predict regulated ontology lists, “tox lists” and networks related to cardiovascular function and disease.

### Parameters analyzed

With respect to our previous study (Keller et al. 2015), we defined ventricle- to body-weight ratio, conventional echocardiographic parameters of systolic left-ventricular (LV) function (ejection fraction, LV end-systolic volume, LV end-diastolic volume, LV end-systolic length), survival time, mRNA-expression levels of *Gnai2*, *Gnai3*, *Nppa*, *Nppb*, *Ryr2*, *Pln*, and *Tnni3*, as well as protein expression levels of Gα_i2_ and Gα_i3_ as primary parameters. We furthermore included left-ventricular global longitudinal strain (GLS), isovolumic relaxation time (IVRT), and the ratio of E’ and A’ (early and late ventricular relaxation velocity) as additional echocardiographic parameters. GLS, derived from speckle tracking-based echocardiography, is a sensitive parameter for early detection of LV systolic and diastolic dysfunction and has been shown to be an independent predictor of all-cause mortality in (human) heart failure with reduced ejection fraction (Sengeløv et al. 2015; de Lucia et al. 2019). IVRT and the E’ to A’ ratio are sensitive indicators of diastolic function (Alex et al. 2018; Schnelle et al. 2018). Furthermore, fibrotic alterations were quantified as percentage of tissue area in ventricular slices. Western blots were performed to reveal the level of phospholamban expression and the ratio of phosphorylated to total Akt. Levels of protein expression and phosphorylation were furthermore obtained by mass spectrometry done with cardiomyocyte homogenates. Non-primary parameters were obtained and analyzed with an exploratory intention.

### Performance of experiments and data analysis

Animals have not explicitly been chosen for a specific experiment or a specific date in a prospective manner. Thus, sequence of investigation was by chance due to availability of an animal at an appropriate age. Sequence of analysis was by chance, too. We did not apply specific methods for randomization of the sequence of experiments or analyses or for blinding of the experimenters. Thus, the analyses were neither specifically blinded nor actively unblinded. Knowledge of the genotype to the investigator may therefore have occurred by chance in individual cases.

### Data presentation and statistical analysis

Data are depicted as scatter plots and reported as mean ± standard deviation (SD) in the text. Scatter plots were created using GraphPad Prism and show median and interquartile range or mean ± SD. We performed ANOVAs that (if significant) were followed by Bonferroni-corrected post-tests comparing all groups with respect to most primary parameters (“Performance of experiments and data analysis” section). For mRNA as well as PLN protein expression, we applied Holm-Šídák post-tests referring to age-matched wildtype littermates. In addition, β_1_-tg and β_1_-tg/Gα_i3_^−/−^ mice were compared here. In case of *Gnai3* mRNA, wildtype and β_1_-tg mice were compared by non-parametric Mann–Whitney test. Due to data distribution, log10 values of 2^−ΔΔCt^ were analyzed with respect to ANP and BNP mRNA expression. Due to the small number of samples, we applied the pair wise fixed reallocation randomization test^©^ using the REST-2009® software for mRNA expression in mice at 300 days of age (Pfaffl 2002). The distribution of EF and IVRT values within two groups was compared using a two-sided Fisher’s exact test. Sample size estimation for echocardiography was performed a priori using G*Power 3.1.9.2 software (Heinrich Heine Universität Düsseldorf, Germany). In general, sample sizes were not increased after reviewing the corresponding data, except for qPCR, where the sample size was increased from *n* = 4 to *n* = 6–12 per group during a process of manuscript revision. Survival times are reported as mean and 95% confidence interval (CI). Survival analysis was performed by Kaplan–Meier estimation followed by the log-rank test. Throughout, we considered *p* values < 0.05 as indicating statistically significant differences in confirmatory analyses, i.e., analyses of the primary parameters according to our main scientific questions (see “Parameters analyzed” and “Introduction”). In figures, asterisks then indicate *p* values below 0.05 (*) and 0.01 (**), respectively. If statistical tests were applied with an exploratory intent (see “Parameters analyzed”), *p* values are given as numbers down to 0.001, but not indicated by asterisks. Statistical approaches have been specified a priori, except for the analysis of distribution of EF and IVRT values (“Gα_i3_ deficiency reduces risk of ventricular dysfunction in β_1_-tg mice” section) that was applied due to the apparent partial overlap of data from β_1_-tg and β_1_-tg/Gα_i3_^−/−^ mice, respectively, and the calculation of Cohen’s *d* affects sizes that was done using the Psychometrica online effect size calculators (Table S3) (Lenhard and Lenhard 2016). From some animals/tissue probes, we obtained more than one parameter. No particular statistical approach was taken to account for this.

## Results

### Gα_i3_ deficiency prolongs survival time in β_1_-tg animals

In order to get insights into the individual role of the Gα_i3_ protein in murine cardiomyopathy, mice overexpressing the cardiac β_1_-adrenoceptor but globally lacking the Gα_i3_ protein (β_1_-tg/Gα_i3_^−/−^) were compared to β_1_-adrenoceptor overexpressing (β_1_-tg), Gα_i3_-deficient (Gα_i3_^−/−^), and wildtype mice. In all mouse lines used, the distribution of genotypes followed Mendel’s rule and that of sex was almost equal (48% male and 52% female). Mice showed no obvious physical phenotype and normal behavior. Deaths occurred suddenly in all groups, at best preceded sporadically by (unspecific) symptoms, e.g., reduced ingestion, striking behavior, or impaired movement.

**Fig. 1:**
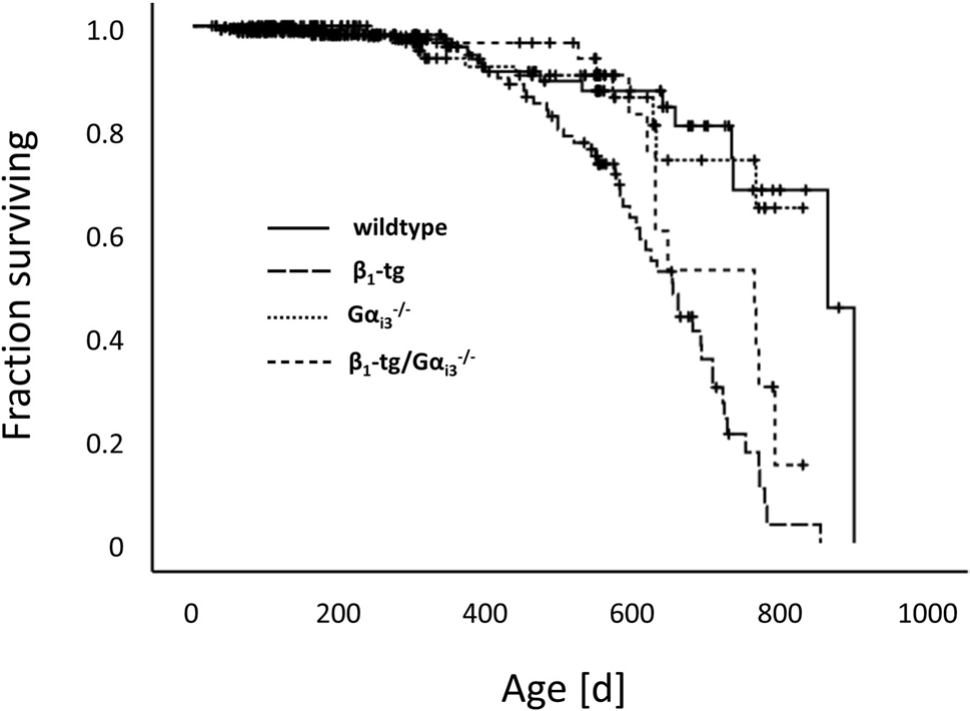
Life span of β_1_-tg mice is significantly shortened compared to wildtype, Gα_i3_^−/−^, as well as β_1_-tg/Gα_i3_^−/−^ mice. Total numbers of mice included in our analysis were 408, 262, 154, and 82 for wildtype, β_1_-tg, Gα_i3_^−/−^, and β_1_-tg/Gα_i3_^−/−^, respectively. Numbers of spontaneous deaths during the period of observation were 17, 52, 11, and 13, respectively. Kaplan-Meyer estimation and log-rank test were applied. Vertical ticks indicate censored events

Survival is a major outcome parameter of heart-failure studies, and in our previous study, we found that Gα_i2_ deficiency caused a significantly shortened lifetime of β_1_-tg mice (Keller et al. 2015). In the current study, we followed the survival of the mice up to a maximum age of 880 days. Kaplan-Meyer estimation and log-rank tests revealed that the mean survival time of β_1_-tg mice was 624 days (95% CI: 592–655), significantly shorter than that of all other genotypes (Fig. 1). Importantly, the concomitant absence of Gα_i3_ increased the mean life span of β_1_-tg mice to 696 days (644–747), which was no longer statistically different from the survival time of 789 days for wildtype (739–840) and 742 days for Gα_i3_^−/−^ mice (693–790), respectively.

In summary, the life span of β_1_-tg/Gα_i3_^−/−^ mice was significantly longer than that of β_1_-tg mice and was not statistically different from that of wildtype littermates, whereas in a previous study, the absence of Gα_i2_ shortened the life expectancy of β_1_-tg mice.

### Study of animals at an age of 300 days

Given that Gα_i2_ deficiency already showed adverse effects in 300-day-old β_1_-tg mice (Keller et al. 2015), we first focused on effects of Gα_i3_ deficiency at this age.

#### ***No cardiac hypertrophy or dysfunction at an age of 300 days***

Survival rates of wildtype, Gα_i3_^−/−^, β_1_-tg, and β_1_-tg/Gα_i3_^−/−^ were similar at an age of 300 days (see Fig. 1). Ventricle-to-body-weight ratio and echocardiographic parameters of ventricular function were comparable in all groups (Table 1). Only the heart rate was significantly increased in β_1_-tg mice, which was even more pronounced in β_1_-tg/Gα_i3_^−/−^ animals. ANP (*Nppa*) and especially BNP (*Nppb*) are useful markers of cardiac hypertrophy and heart failure. Neither *Nppa* nor *Nppb* mRNA levels showed statistically significant alterations in β_1_-tg/Gα_i3_^−/−^ or Gα_i3_^−/−^ ventricles compared to wildtypes (Table 2). However, similar to our previous findings (Keller et al. 2015), *Nppb* mRNA levels were significantly increased in ventricles of β_1_-tg mice already at this younger age (304 ± 145% of wildtype levels, *p* < 0.05; Table 2). Expression levels of ryanodine receptor type 2 (*Ryr2*), phospholamban (*Pln*), and Troponin I (*Tnni3*) mRNA were similar in the ventricles of all genotypes (Table 2).

**Table 1:** Ventricle- to body-weight ratios and echocardiographic data at an age of 300 days (mean ± SD, number of animals in brackets)

Genotype	Wildtype	β_1_-tg	Gα_i3_^−/−^	β_1_-tg/Gα_i3_^−/−^
Ventricle to body-weight ratio (%)	0.44 ± 0.09 (19)	0.43 ± 0.08 (16)	0.45 ± 0.07 (16)	0.42 ± 0.04 (18)
Ejection fraction (%)*	60 ± 6 (8)	52 ± 3 (10)	52 ± 8 (8)	58 ± 6 (9)
Left-ventricular end-systolic volume [µL]	28 ± 9 (8)	34 ± 11 (10)	35 ± 13 (8)	24 ± 6 (9)
Left-ventricular end-diastolic volume [µL]	70 ± 18 (8)	71 ± 19 (10)	71 ± 18 (8)	56 ± 7 (9)
Left-ventricular end-systolic length [mm]	7.2 ± 0.5 (8)	7.8 ± 0.6 (10)	7.1 ± 0.6 (8)	7.2 ± 0.8 (9)
Myocardial performance index	0.72 ± 0.11 (8)	0.91 ± 0.32 (10)	0.74 ± 0.14 (8)	0.71 ± 0.18 (9)
Global longitudinal strain (%)	− 21.5 ± 2.4 (4)	− 18.5 ± 5.9 (8)	− 25.9 ± 5.1 (4)	− 22.1 ± 8.7 (7)
*E* ‘/*A*‘	1.4 ± 0.6 (5)	1.4 ± 0.7 (8)	1.6 ± 0.6 (5)	2.4 ± 1.4 (8)
Heart rate [min^−1^]*	448 ± 53 (8)	521 ± 52^**#**^ (10)	492 ± 69 (8)	588 ± 39^#,§^ (9)

For each parameter, a one-way ANOVA was performed (*p < 0.05). ^**#**^*p* < 0.05 vs. wildtype and ^§^*p* < 0.05 *vs.* β_1_-tg in Bonferroni-corrected post-tests between all groups

**Table 2:** qPCR data on ventricular mRNA expression at an age of 300 days (% of wildtype; mean ± SD)

Genotype (N)	β_1_-tg (3)	Gα_i3_^−/−^ (3)	β_1_-tg/Gα_i3_^−/−^ (3)
*Gnai2*	96 ± 23	85 ± 28	70 ± 45
*Gnai3*	179 ± 81	0 ± 0*	0 ± 0*
*Nppa* (ANP)	351 ± 264	162 ± 16	137 ± 160
*Nppb* (BNP)	304 ± 145*	117 ± 73	104 ± 40
*Ryr2*	115 ± 36	98 ± 21	98 ± 31
*Pln*	115 ± 21	116 ± 12	98 ± 21
*Tnni3*	94 ± 18	115 ± 14	87 ± 15

^*^p < 0.05 vs. wildtype in a pair wise fixed reallocation randomization test^©^ using the REST-2009® software

*Gnai2* mRNA expression levels were dominant over *Gnai3* in wildtype ventricles (data not shown) and similar to wildtype mice in ventricular tissue from β_1_-tg, β_1_-tg/Gα_i3_^−/−^ or Gα_i3_^−/−^ mice (Table 2). As expected, *Gnai3* mRNA expression was not detectable in ventricles of Gα_i3_^−/−^ and β_1_-tg/Gα_i3_^−/−^ mice. Western blot analysis confirmed these findings on the protein level (not shown).

In summary, neither ventricular hypertrophy nor dysfunction was observed in any of the investigated groups at the age of 300 days. In contrast to lack of Gα_i2_ (Keller et al. 2015), a detrimental effect of Gα_i3_ deficiency in β_1_-tg mice at this age is unlikely.

### Study of animals at an age of 550 days

In line with increased mortality of β_1_-tg mice at more advanced ages, we next examined animals at 550 days of age. Effect sizes obtained by comparing wildtype with β_1_-tg, Gα_i3_^−/−^ and β_1_-tg/Gα_i3_^−/−^ and β_1_-tg with β_1_-tg/Gα_i3_^−/−^ mice at this age can be taken from supplemental Table 3 (Table S3).

#### ***Examination of ventricular hypertrophy and fibrosis***

**Fig. 2:**
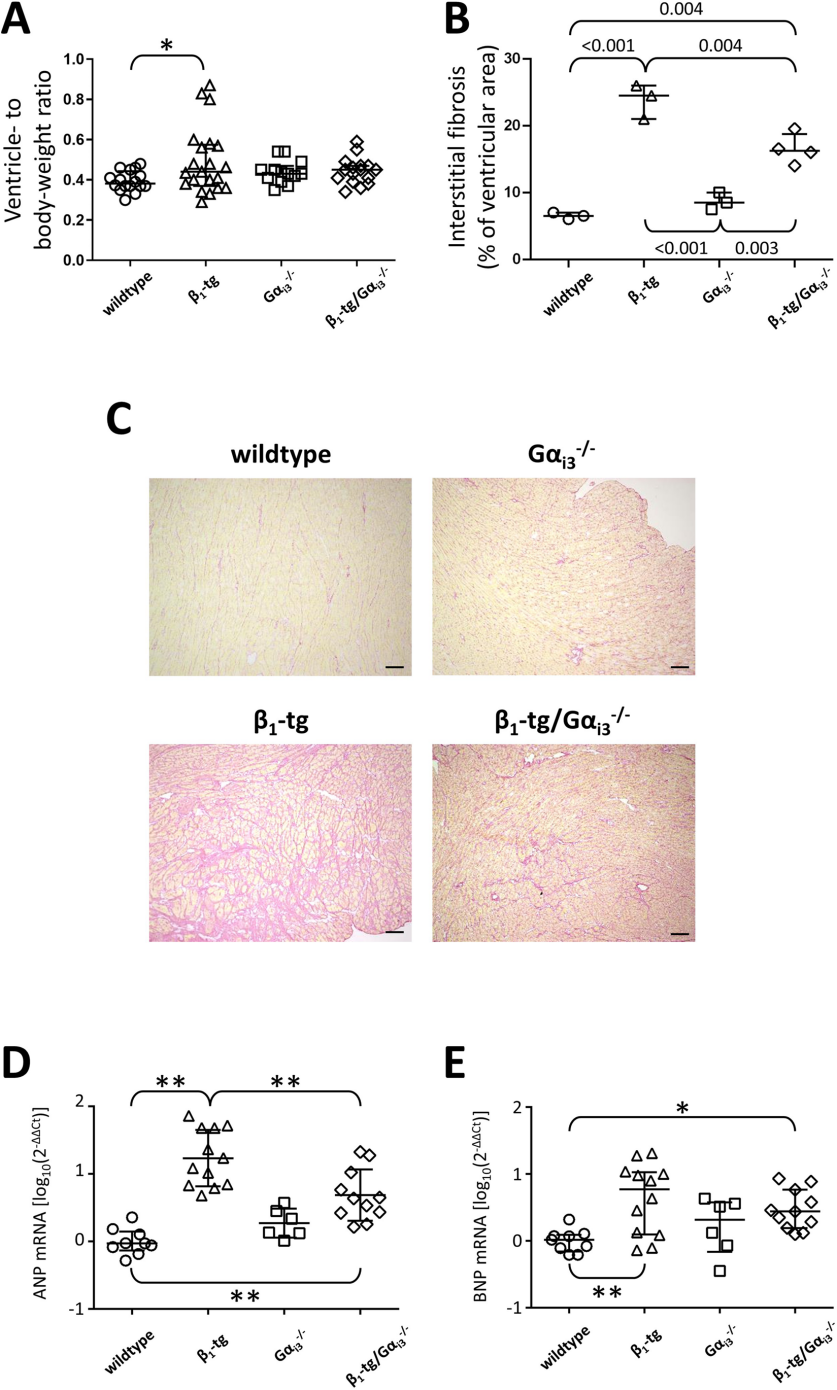
Ventricular hypertrophy, fibrosis, and vastly increased hypertrophy markers in β_1_-tg mice at an age of 550 days. **A** Ventricle- to body-weight ratios were calculated for 16 wildtype, 21 β_1_-tg, 13 Gα_i3_^−/−^, and 16 β_1_-tg/Gα_i3_^−/−^ mice. **B**, **C** Sirius Red staining was applied to ventricular cryo-Sects. (3 ventricles from wildtype, β_1_-tg, and Gα_i3_^−/−^, each, and 4 ventricles from β_1_-tg/Gα_i3_^−/−^ mice), and interstitial fibrosis was quantified as percentage of total tissue area. Ventricles of β_1_-tg mice demonstrated an increase in fibrotic area that was significantly attenuated by lack of Gα_i3_. **A**, **B** All groups were compared with each other. **C** Representative mid-ventricular cardiac sections from wildtype, β_1_-tg, Gα_i3_^−/−^, and β_1_-tg/Gα_i3_^−/−^ mice after Sirius Red staining. Scale bars: 100 µm. **D**, **E** mRNA expression of ANP (*Nppa*, **D**) and BNP (*Nppb*, **E**) corresponds to the extent of fibrosis observed in mice overexpressing β_1_-adrenoceptors. Data from 9 wildtype, 12 β_1_-tg, 6 Gα_i3_^−/−^, and 11 β_1_-tg/Gα_i3_^−/−^ mice were obtained in triplicate each. Groups were compared with age-matched wildtypes. In addition, β_1_-tg and β_1_-tg/Gα_i3_^−/−^ mice were compared. Due to data distribution, log10 values of 2^−^^ΔΔCt^ were analyzed. Scatter plots with median and interquartile range are depicted (**A**, **B**, **D**, and **E)**. * and **: *p* < 0.05 and *p* < 0.01 in post-tests following ANOVA (**A**, **D**, and **E**). Fibrotic areas were compared with an exploratory intention, and thus, exact *p* values are given if < 0.05 (**B**)

At an age of 550 days, β_1_-tg mice showed ventricular hypertrophy indicated by a statistically significant increase in the mean ventricle-to-body weight ratio compared to wildtype mice (Fig. 2A). No such effect was seen if β_1_-tg mice were lacking Gα_i3_. Mice overexpressing the β_1_-AR developed ventricular fibrosis, which was significantly less pronounced in β_1_-tg/Gα_i3_^−/−^ mice (Fig. 2B, C). Fitting to this, mRNA levels of the hypertrophy markers ANP (*Nppa*) and BNP (*Nppb*) were significantly increased in β_1_-tg ventricles, but to a lesser extent when Gα_i3_ was absent (*Nppa*: 2578 ± 2323% vs. 705 ± 688%; *Nppb*: 744 ± 688% vs. 363 ± 260%) (Fig. 2D, E).

These findings of hypertrophy and fibrosis may be related to ventricular dysfunction in β_1_-tg mice on the one hand and to protective effects of Gα_i3_ deficiency on the other. Therefore, we describe below our findings on left ventricular function obtained by echocardiography.

#### ***Gα***_***i3***_*** deficiency reduces risk of ventricular dysfunction in β***_***1***_***-tg mice***

**Fig. 3:**
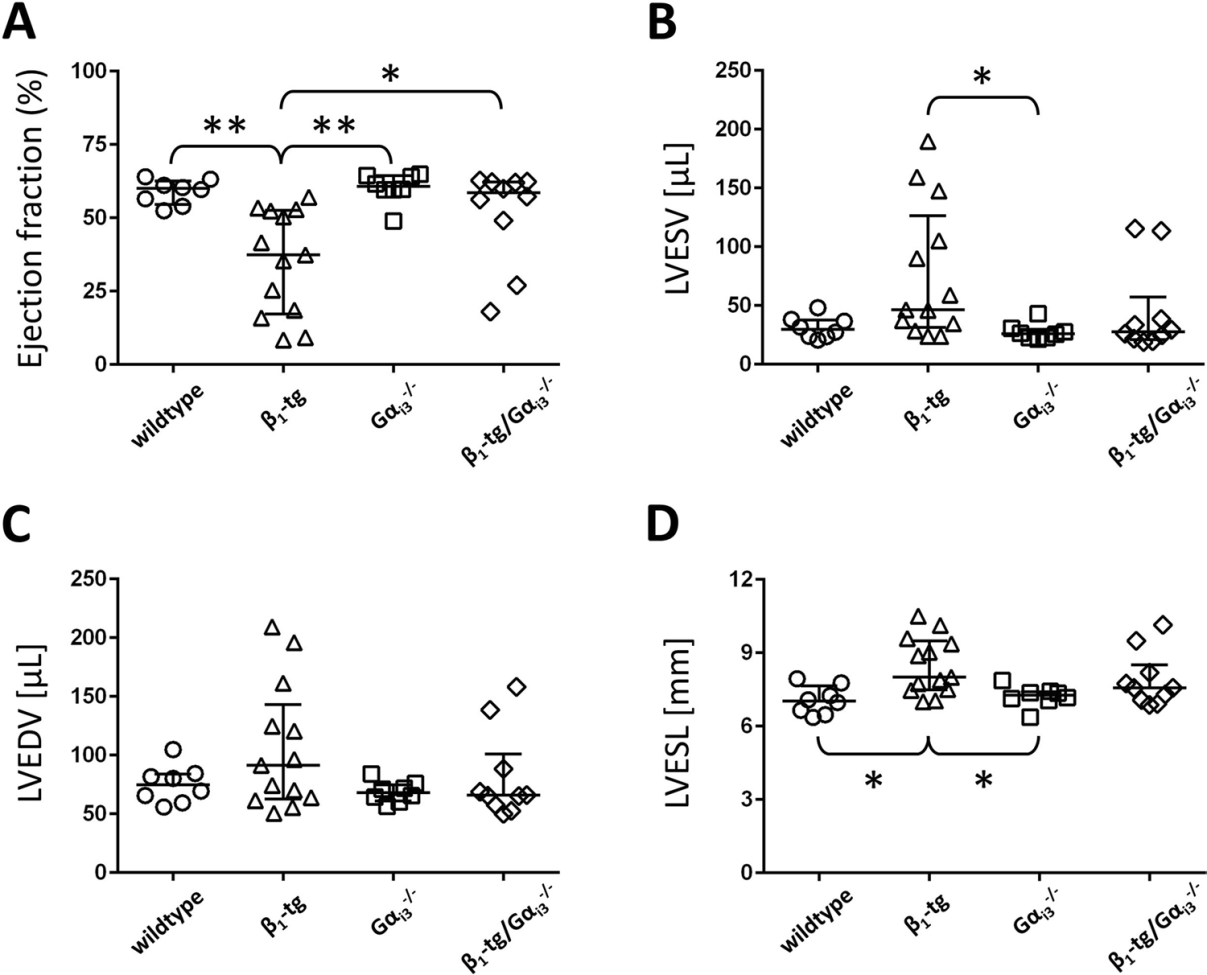
Ventricular dysfunction in β_1_-tg mice at an age of 550 days. As echocardiographic parameters representing systolic function, ejection fraction (**A**), left-ventricular (LV) end-systolic volume (**B**), LV end-diastolic volume (**C**), and LV end-systolic length (**D**) are shown. Echocardiographic data were obtained from 8 wildtype, 13 β_1_-tg, 8 Gα_i3_^−/−^, and 10 β_1_-tg/Gα_i3_^−/−^ mice. Scatter plots with median and interquartile range are depicted. If the ANOVA indicated statistically significant differences, it was followed by Bonferroni-corrected post-tests between all groups. * and **: *p* < 0.05 and *p* < 0.01

β_1_-tg mice showed ventricular dysfunction indicated by a statistically significant decrease of the ejection fraction (EF: 35 ± 18%, *n* = 13) and an increase of the mean LV end-systolic volume, the end-diastolic volume, and the end-systolic length (Fig. 3). In contrast, the EF of β_1_-tg/Gα_i3_mice^−/−^ (52 ± 16%, *n* = 10) was significantly higher than that of β_1_-tg mice and similar to wildtype (59 ± 4%, *n* = 8) and Gα_i3_^−/−^ (60 ± 5%, *n* = 8). Reduced EF levels were also found in a few β_1_-tg/Gα_i3_^−/−^ mice, but significantly less frequently than in β_1_-tg mice, relative to the 95% CI of age-matched wildtypes (3 out of 10 vs. 12 out of 13, *p* = 0.003).

**Fig. 4:**
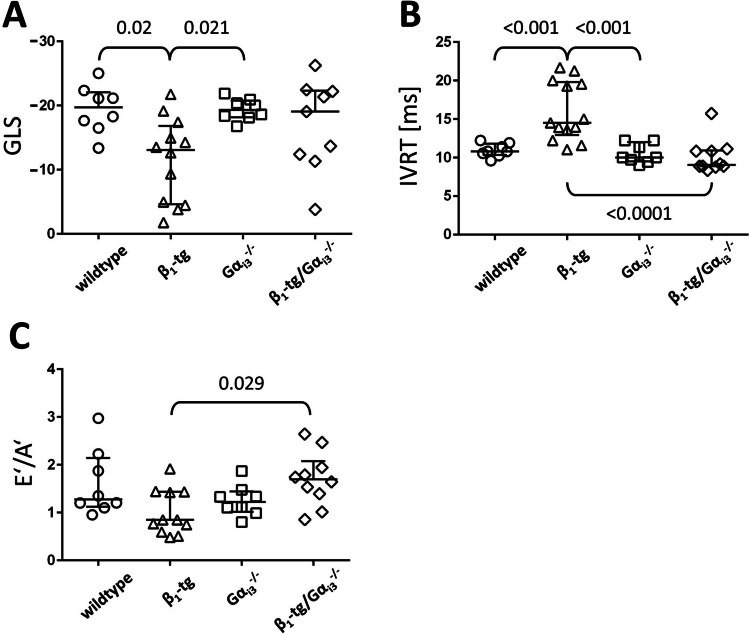
Exploratory echocardiographic analyses reveal impaired diastolic ventricular function of β_1_-tg mice at an age of about 550 days. Global longitudinal strain (GLS) (**A**), isovolumic relaxation time (IVRT) (**B**), and *E*’ to *A*’ ratio (*E*’/*A*’) (**C**) were analyzed as parameters of global (GLS), or diastolic function (IVRT, *E*’/*A*’), respectively. Group sizes were 8 for wildtype and Gα_i3_^−/−^, 12 (GLS), 11 (*E*’/*A*’) and 13 (IVRT) for β_1_-tg, and 9 (GLS) and 10 (*E*’/*A*’ and IVRT) for β_1_-tg/Gα_i3_^−/−^ mice, respectively. Scatter plots with median and interquartile range are shown. If the ANOVA indicated statistically significant differences, it was followed by Bonferroni-corrected post-tests between all groups. Analysis was done with an exploratory intention, and thus, exact *p* values are given if < 0.05

LV global longitudinal strain (GLS), an independent predictor of all-cause mortality in (human) heart failure with reduced ejection fraction (Sengeløv et al. 2015), was significantly impaired in β_1_-tg mice in an exploratory analysis (Fig. 4A). In contrast, β_1_-tg/Gα_i3_^−/−^ mice did not differ from wildtype and Gα_i3_^−/−^ mice. Regarding diastolic LV function, we analyzed the isovolumic relaxation time (IVRT). We observed a statistically significant impairment of IVRT in β_1_-tg mice (Fig. 4B), while the absence of Gα_i3_ normalized this parameter to wildtype and Gα_i3_^−/−^ values. Furthermore, the impairment of the *E*’ to *A*’ ratio in β_1_-AR overexpressing mice was no longer observed in β_1_-tg mice lacking Gα_i3_ (Fig. 4C).

Taken together, Gα_i3_ deficiency reduced the risk of both systolic and diastolic LV dysfunction in β_1_-tg mice.

#### ***Ventricular expression of G***_***i***_*** proteins and Akt***

**Fig. 5:**
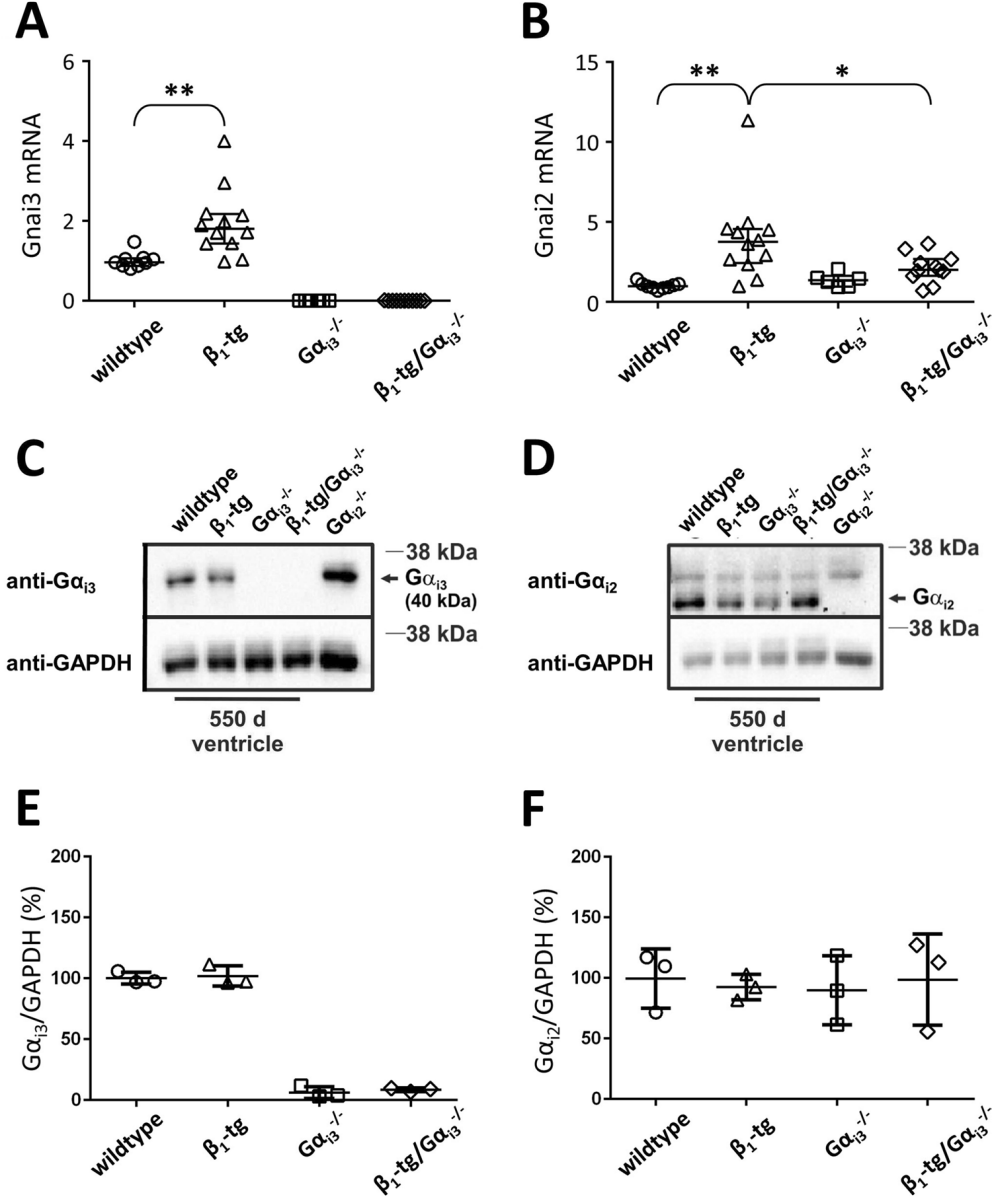
G_i_ expression at the mRNA and the protein level. Relative expression of **A** Gα_i3_ (*Gnai3*) and **B** Gα_i2_ mRNA (*Gnai2*) is depicted as 2^−ΔΔCt^ referring to wildtype controls. qPCR data from 550-day-old wildtype (*n* = 9), β_1_-tg (*n* = 12), Gα_i3_^−/−^ (*n* = 6), and β_1_-tg/Gα_i3_^−/−^ (*n* = 11) mice were obtained in triplicate each. **C**, **D** Representative Western blots of ventricle homogenates isolated from 550-day-old wildtype, β_1_-tg, Gα_i3_^−/−^, and β_1_-tg/Gα_i3_^−/−^ mice. To verify antibody specificity, ventricle homogenates from Gα_i2_-deficient mice were loaded. Gα_i3_-protein expression (**C**) is completely absent in Gα_i3_^−/−^ and β_1_-tg/Gα_i3_.^−/−^ ventricles, while not obviously altered in β_1_-tg ventricles. Gα_i2_ protein (**D**) is detectable in ventricles isolated from any genotype. For exemplary full Western blots, see supplemental Figure S2. **E**, **F** Statistical analysis of Gα_i3_ and Gα_i2_ protein expression patterns using GAPDH as loading control. For Western blot analysis, ventricles from three animals per genotype were analyzed in three independent experiments. Scatter plots with median and interquartile range (**A**, **B**) or mean values ± SD are shown (**E**, **F**). * and **: *p* < 0.05 and *p* < 0.01 in a Mann–Whitney test (**A**, **E**) or in post-tests performed if an ANOVA indicated significant differences (**B**). ANOVA of data on protein expression indicated no difference (**F**)

In wildtypes at an age of 550 days, ventricular mRNA levels of *Gnai2* transcripts were confirmed to be still dominant over *Gnai3* (data not shown). As expected, *Gnai3* mRNA was not detectable in ventricles of Gα_i3_^−/−^ and β_1_-tg/Gα_i3_^−/−^ mice (Fig. 5A). Compared to wildtype mice, there was a statistically significant increase of *Gnai* mRNA in β_1_-tg ventricles (*Gnai2*: 397 ± 265%, *Gnai3*: 196 ± 88%; Fig. 5A, B). There was no statistically significant alteration of *Gnai2*-mRNA expression in ventricular tissue from β_1_-tg/Gα_i3_^−/−^ or Gα_i3_^−/−^ mice compared to wildtype mice (Fig. 5B). We determined protein expression to corroborate our mRNA data. Except for the absence of Gα_i3_ in the ventricles of Gα_i3_^−/−^ and β_1_-tg/Gα_i3_^−/−^ mice (Fig. 5C, E), no difference in Gα_i3_ or Gα_i2_ expression was found between groups at the protein level (Fig. 5C–F; for uncropped Western blots, see Figure S2).

**Fig. 6:**
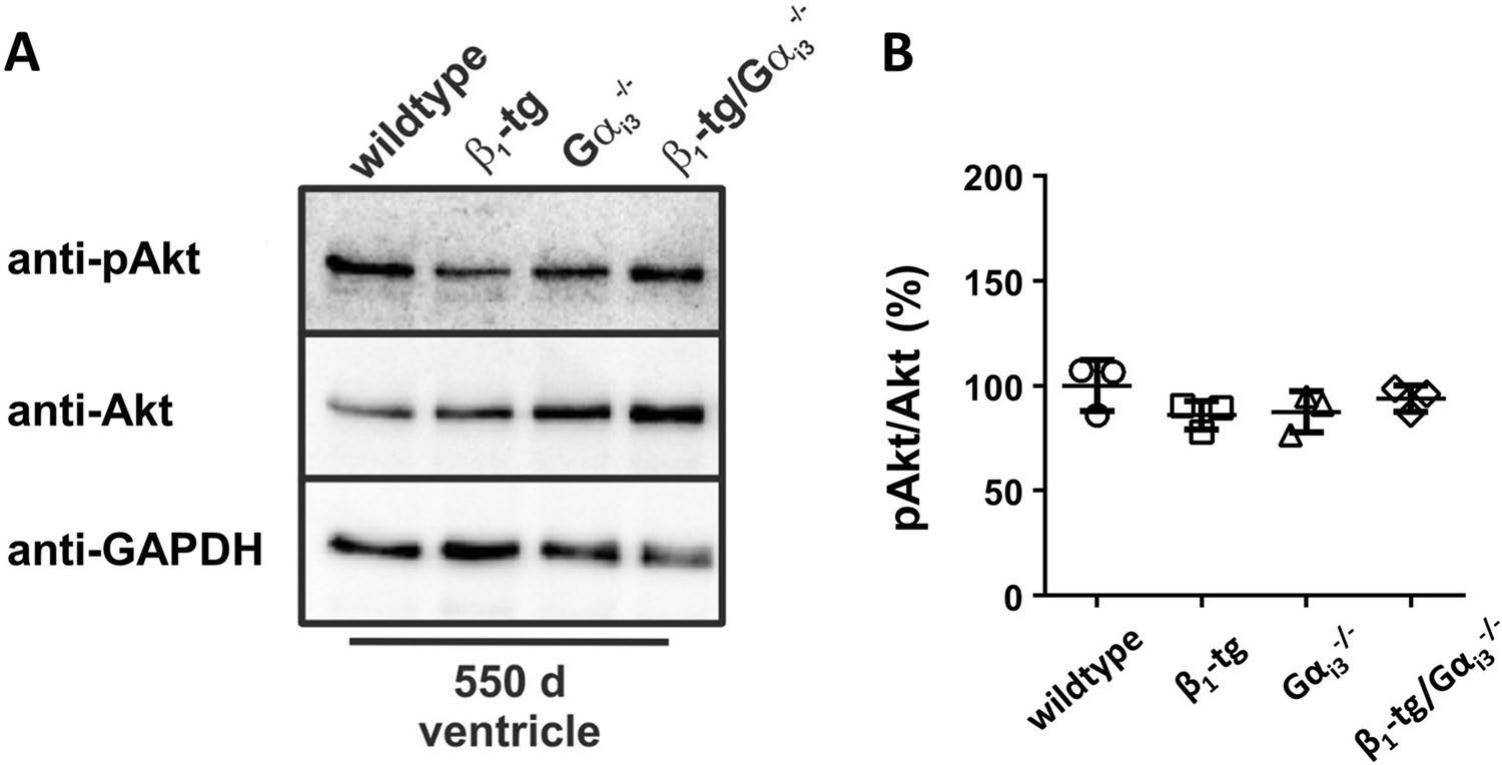
**A** Akt and phosphorylated Akt (pAkt) were detected using specific antibodies in Western blots of ventricle homogenates obtained from 550-day-old wildtype, β_1_-tg, Gα_i3_^−/−^, and β_1_-tg/Gα_i3_^−/−^ mice (*n* = 3 each). **B** ANOVA did not reveal statistically significant differences of pAkt/Akt ratios (mean values ± SD)

Akt activation has been linked to cardiomyopathy, and studies from other tissues indicated isoform-specific modulation by G_i_ proteins. We thus analyzed expression of phosphorylated Akt protein. Western blots, however, revealed no obvious differences when comparing pAkt/Akt ratios in ventricles of wildtype, β_1_-tg, Gα_i3_^−/−^ and β_1_-tg/Gα_i3_^−/−^ mice (Fig. 6). Consistent with this, quantitative proteomics analyses demonstrated no changes in Akt expression or Akt phosphorylation (see the “Proteomics and pathway analyses” section).

In summary, both *Gnai2* and *Gnai3* mRNA levels were increased in 550-day-old β_1_-tg mice while we found no change at the protein level. Akt phosphorylation was not obviously affected by either β_1_-AR overexpression or Gα_i3_ deficiency.

#### ***Ventricular expression of the PKA targets ryanodine receptor 2, phospholamban, and cardiac troponin I***

**Fig. 7:**
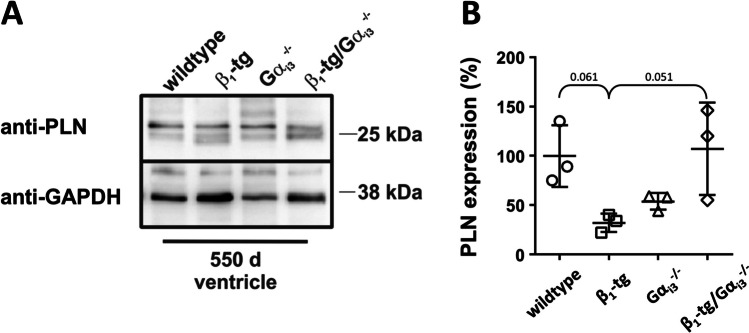
**A** Phospholamban (PLN) was detected using a specific antibody in Western blots of ventricle homogenates obtained from 550-day-old wildtype, β_1_-tg, Gα_i3_^−/−^, and β_1_-tg/Gα_i3_^−/−^ mice (*n* = 3 each). **B** ANOVA indicated statistically significant differences in PLN expression levels normalized to GAPDH (*p* = 0.036), mainly due to a decrease in β_1_-tg mice (*p* = 0.061 vs. wildtype and *p* = 0.051 vs. β_1_-tg/Gα_i3_^−/−^). Mean values ± SD are depicted

**Fig. 8:**
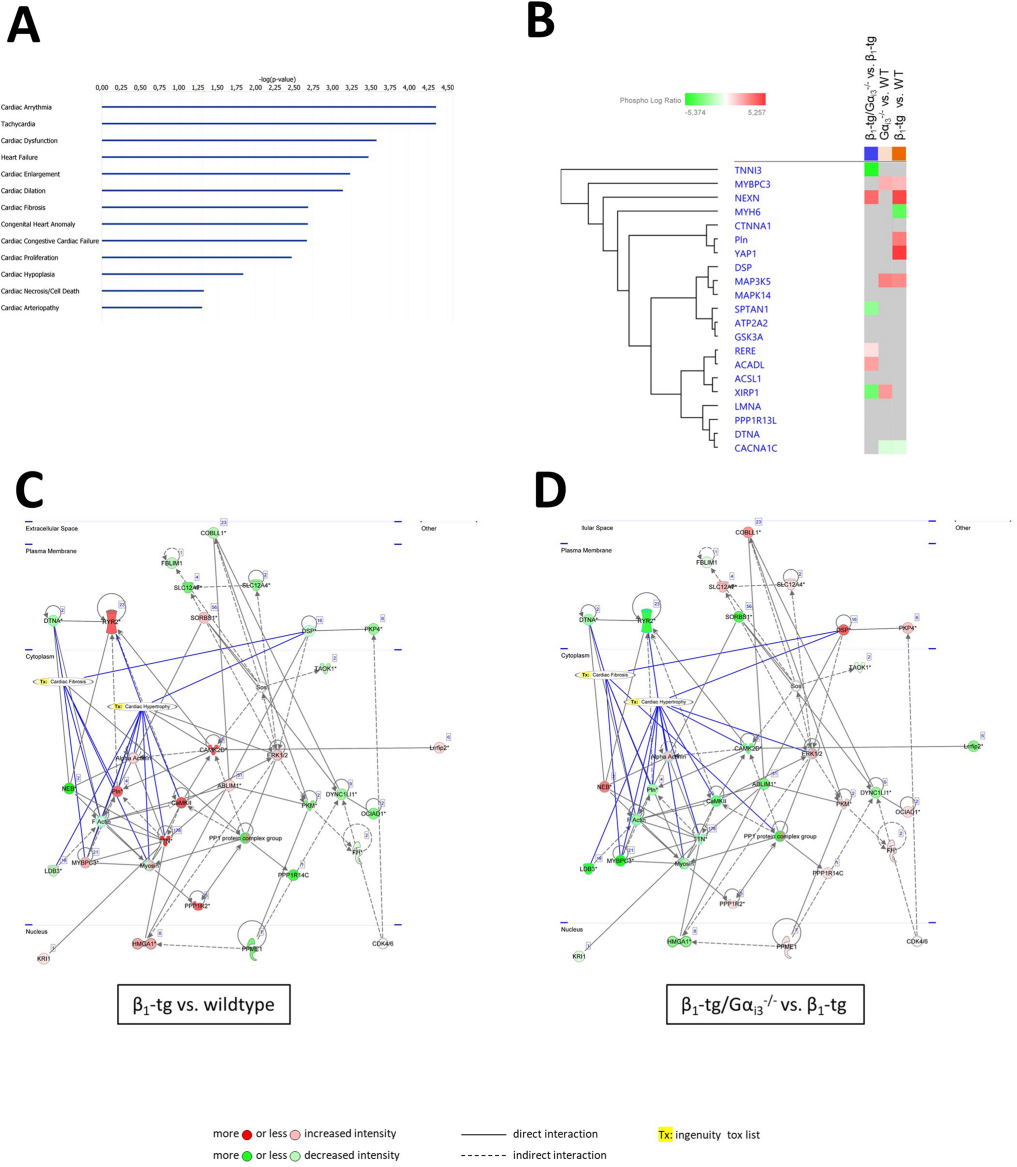
Protein phosphorylation levels in ventricular myocytes (three per genotype) were obtained by mass spectrometry and fed into a so-called ingenuity pathway analysis (IPA), an algorithm-based analysis that uses the QIAGEN knowledge base to identify differences in signaling pathways. **A** As indicated by − (log *p*) values, IPA revealed significant differences between β_1_-tg and β_1_-tg/Gα_i3_^−/−^ mice in activation of disease-associated pathways. **B** Heatmap representing differential phosphorylation of proteins assigned to the “cardiac fibrosis pathway.” Activation *Z*-scores referring to the respective genotypes compared are color-coded (top row) from blue (“lower activity”) to orange (“higher activity”). An increase in the phosphorylation of a particular protein is shown in red, a decrease in green (lower rows). Gray color indicates comparisons that did not reveal statistical significance (*p* > 0.05). When comparing β_1_-tg with wildtype (**C**), and β_1_-tg/Gα_i3_^−/−^ with β_1_-tg mice (**D**), the top scoring IPA network indicated differences related to cardiac dysfunction and cardiovascular disease. Red indicates that in the genotype mentioned first, a protein is more phosphorylated relative to the comparator; green stands for reduced phosphorylation. For example, phospholamban (PLN) phosphorylation is increased in β_1_-tg compared to wildtype myocytes, while it is reduced in β_1_-tg/Gα_i3_^−/−^ compared to β_1_-tg mice. Relationships between proteins (nodes) and heart diseases (cardiac fibrosis, cardiac hypertrophy) are indicated (*Tx*, toxicity-related (“tox”) lists)

mRNA levels of the PKA phosphorylation targets ryanodine receptor 2 (*Ryr2*), phospholamban (*Pln*), and cardiac troponin I (*Tnni3*) did not differ between the four genotypes (Figure S3). We furthermore analyzed phospholamban expression by Western blotting (Fig. 7). An ANOVA indicated significant differences (*p* = 0.036), mainly due to decreased PLN levels in ventricles of β_1_-tg mice (32 ± 9%) compared to wildtype (100 ± 31%; *p* = 0.061) and β_1_-tg/Gα_i3_^−/−^ mice (107 ± 47%; *p* = 0.051). Of interest, proteomics analysis furthermore revealed statistically significant alterations of PLN phosphorylation in β_1_-tg ventricular myocytes, which were not seen in β_1_-tg/Gα_i3_^−/−^ mice (see the “Proteomics and pathway analyses” section and Fig. 8C, D).

In summary, Western blots indicated reduced PLN expression in ventricles of β_1_-tg, but not β_1_-tg/Gα_i3_^−/−^ mice at the age of 550 days. *Ryr2*, *Pln*, and *Tnni*3 mRNA levels appeared to be unaffected.

### Proteomics and pathway analyses

Protein and protein phosphorylation levels in ventricular myocytes from wildtype, β_1_-tg, Gα_i3_^−/−^, and β_1_-tg/Gα_i3_^−/−^ mice (*n* = 3 animals each, age: 200 ± 56 days) were determined by mass spectrometry. We did not detect any change in Akt expression or phosphorylation in agreement with the data from Western blot analysis (cp. Fig. 6). For phospholamban, however, we found a statistically significant increase in phosphorylation at Ser16 (*p* = 0.007) and Ser17 (*p* = 0.013) in β_1_-tg compared to wildtype ventricular myocytes that was not seen in β_1_-tg/Gα_i3_^−/−^ myocytes.

For further analysis, we fed the protein phosphorylation data into a so-called ingenuity pathway analysis (IPA). IPA is an algorithm-based analysis that uses the QIAGEN knowledge base to identify differences in signaling pathways. When comparing our β_1_-tg and β_1_-tg/Gα_i3_^−/−^ target genotypes, analyses revealed statistically significant differences associated with multiple cardiac disorders and diseases (Fig. 8A). IPA suggested activation of the predefined protein ontology list “cardiac fibrosis” in β_1_-tg compared to wildtype ventricles, while activity was reduced in β_1_-tg/Gα_i3_^−/−^ compared with data from β_1_-tg mice (Fig. 8B). To provide further evidence for possible mechanisms, the proteins detected in our probes were mapped to the networks available in the underlying QIAGEN database and then scored using a network score based on *p* values obtained in Fisher’s exact test. For the sample-specific network that achieved the highest score, β_1_-tg and β_1_-tg/Gα_i3_^−/−^ myocytes showed some differences in phosphorylation of interacting proteins linked to “tox lists” such as “cardiac fibrosis” and “cardiac hypertrophy” (Fig. 8C, D). For example, PLN, RYR2, and calmodulin kinase II (CaMK II) phosphorylation is seen to be increased in β_1_-tg compared to wildtype myocytes (color-coded red in Fig. 8C), whereas it is lower in β_1_-tg/Gα_i3_^−/−^ compared with β_1_-tg mice (color-coded green in Fig. 8D).

Taken together, proteomics analyses showed that in β_1_-tg ventricular myocyte phospholamban phosphorylation levels were significantly increased. Pathway and network analyses based upon protein phosphorylation indicated opposite patterns in β_1_-tg compared to β_1_-tg/Gα_i3_^−/−^ mice with respect to cardiac dysfunction and disease. These results are in good agreement with our in vivo data of ventricular dysfunction and our in vitro data such as ventricular fibrosis or increased expression of hypertrophy markers.

## Discussion

Given the previously shown detrimental effects of a Gα_i2_ deficiency in mice with a cardiac overexpression of β_1_-AR (Keller et al. 2015), we now asked for the role of the closely related Gα_i3_ isoform in this murine heart-failure model. Since β_1_-transgenic (β_1_-tg) mice develop progressively impaired cardiac functions accompanied by a significantly shortened life span, this heart-failure model is suitable to test for effects of an additional Gα_i3_ deficiency. We wondered how Gα_i3_ deficiency affects cardiac function and outcome of β_1_-tg mice, i.e., whether it is detrimental, protective, or has no effect.

### G_i_ proteins in β-AR-mediated heart failure

Our current study revealed that the absence of Gα_i3_ in β_1_-AR-overexpressing mice was protective, slowing or even preventing the development of heart failure. In contrast, we previously found that the absence of Gα_i2_ in β_1_-tg mice resulted in a distinct heart-failure phenotype even before it was evident in mice overexpressing only the β_1_-AR (Keller et al. 2015). Thus, the possibility that Gα_i3_ deficiency mimics the Gα_i2_-knockout phenotype in the β_1_-tg model of dilative cardiomyopathy can be excluded. One may hypothesize that the remaining Gα_i_ isoform functionally replaces the missing isoform. Indeed, the absence of one Gα_i_ isoform is often accompanied by upregulation of the remaining one (Wiege et al. 2012; Köhler et al. 2014; Devanathan et al. 2015; Beer-Hammer et al. 2018), although Western blot analyses have been inconsistent regarding an increase of cardiac Gα_i2_ expression in Gα_i3_-deficient mice at the protein level (Gohla et al. 2007; Dizayee et al. 2011; Hippe et al. 2013; Köhler et al. 2014). In the current study, we did not see an upregulation of Gα_i2_ in Gα_i3_-deficient hearts. However, one should keep in mind that cardiac Gα_i2_ expression exceeds that of Gα_i3_ per se. Therefore, we cannot exclude the possibility that Gα_i2_ contributes by functional substitution even in the presence of unchanged (i.e., “normal”) expression levels. On the other hand, (cardiac) Gα_i3_ levels might be generally too low to compensate for Gα_i2_ deficiency. This could explain why we observed adverse effects of Gα_i2_ deficiency in the previous study, although there was a statistically significant increase in Gα_i3_-protein expression (Keller et al. 2015). Unfortunately, due to its embryonic lethality, the Gα_i2/i3_ double knockout mouse model cannot be used to test the assumption that the Gα_i_ isoforms can substitute for each other in the β_1_-tg mouse model (Gohla et al. 2007). At the mRNA level, expression of both Gα_i2_ (*Gnai2*) and Gα_i3_ (*Gnai3*) transcripts appeared to be increased in β_1_-tg ventricles while there was no obvious change at the protein level. In rats treated with isoproterenol, the increase in *Gnai* mRNA transcript levels was significantly more pronounced than the increase in Gα_i_ protein expression (Mende et al. 1992; Eschenhagen et al. 1992a). Thus, we cannot exclude that we have missed an only slight increase of Gα_i_ expression at the protein level.

Although survival is reduced by cardiac overexpression of β_1_-adrenoceptors alone, it has been even worse in β_1_-tg mice lacking Gα_i2_ (Keller et al. 2015). In contrast, we now find that the life span of β_1_-tg mice is significantly increased if they lack Gα_i3_. Although 550-day-old β_1_-tg mice lacking Gα_i3_ showed increased cardiac ANP and BNP mRNA levels compared to wildtype littermates, the ANP increase was significantly lower compared to mice only overexpressing the β_1_-AR. In addition, it appeared to be clearly lower than in Gα_i2_-deficient β_1_-tg mice at an age of 300 days as analyzed in our previous study (Keller et al. 2015). Cardiac overexpression of β_2_-adrenoceptors also leads to cardiac failure, although a significantly higher level of overexpression is required (Liggett et al. 2000). Similar to our recent findings with β_1_-tg mice, lack of Gα_i2_ drastically shortened the lifespan of mice with a cardiac overexpression of the β_2_-AR subtype in another study (Foerster et al. 2003; Keller et al. 2015). Of note, β_2_-tg mice with a homozygous Gα_i2_ knockout were virtually non-viable and already heterozygous Gα_i2_ deficiency reduced life span to a similar extent as did the complete absence of Gα_i2_ on a background of cardiac β_1_-AR overexpression. These findings may reflect the role of G-proteins for either β_1_-AR- or β_2_-AR-mediated signaling: while G_s_ proteins are the cognate interaction partners of β_1_-AR (Xiao et al. 1999; Seyedabadi et al. 2019), it is widely accepted that β_2_-AR couple to both G_s_ and G_i_ proteins (Xiao et al. 1999, 2003). With respect to putative isoform-specific effects of G_i_ proteins, it should be noted that in a mouse model of ischemia–reperfusion-induced cardiac damage, Köhler et al. also found detrimental effects of Gα_i2_ deficiency on the one hand while Gα_i3_ deficiency appeared to be cardioprotective on the other hand (Köhler et al. 2014).

In conclusion, we show that in a mouse model of dilative cardiomyopathy, Gα_i3_ deficiency is beneficial. In contrast, lack of Gα_i2_ was clearly detrimental in previous studies, either in the same heart-failure model of β_1_-AR overexpression, a model of β_2_-AR overexpression or with ischemia–reperfusion as pathophysiological stimulus (Foerster et al. 2003; Köhler et al. 2014; Keller et al. 2015).

### Differences of Gα_i2_- and Gα_i3_-dependent effects at the cellular and subcellular level

Data from neutrophils suggest an interesting difference in Gα_i2_- versus Gα_i3_-mediated signaling: in a study of Kuwano et al., Gα_i2_ deficiency led to an increase, but Gα_i3_ deficiency to a decrease of Akt phosphorylation (Kuwano et al. 2016). Akt has been described to be involved in cardio-protective signaling, while on the other hand, chronic activation of the PI3K/Akt cascade is related to cardiac hypertrophy, and Akt activity was increased in human failing hearts (Haq et al. 2001; Nagoshi et al. 2005). In a previous study, we found no difference between Akt phosphorylation in Gα_i2_- or Gα_i3_-deficient mice, neither under basal conditions nor after treating mice with carbachol (Dizayee et al. 2011). The opposing results of our previous study and that of Kuwano et al. may be explained not only by the various tissues analyzed but the different genetic backgrounds (C57/BL6 and 129/Sv, respectively) which have been associated with phenotypic differences in G_i_-knockout models (Offermanns 1999; Kuwano et al. 2016). Regarding our previous findings on Akt in mice lacking Gα_i2_ or Gα_i3_ (Dizayee et al. 2011), one should bear in mind that carbachol is rather considered a non-pathologic stimulus, and stimulation of muscarinic receptors might be beneficial under pathological conditions, e.g., heart failure (Communal et al. 1999; Olshansky et al. 2008; Lorenz et al. 2009). Although we cannot eventually rule out a change, no obvious differences in ventricular pAkt expression were found in the current study. Given the otherwise pronounced effects on Akt phosphorylation in human heart failure and in heart failure models (Haq et al. 2001; Baba et al. 2003; Miyamoto et al. 2004), it seems at least unlikely that the marked differences in cardiac function and survival in our study can be explained by changes in Akt phosphorylation.

Western blots suggested a reduced PLN expression in β_1_-tg compared to both wildtype and β_1_-tg/Gα_i3_^−/−^ mice. We furthermore used protein expression and phosphorylation data obtained from ventricular myocytes for ingenuity pathway analysis (IPA). IPA has the advantage of a lower risk of bias than manual analysis of the results would have. Our data indicate significant differences between β_1_-tg and β_1_-tg/Gα_i3_^−/−^ mice with respect to intracellular signaling relevant to several cardiac diseases including arrhythmia, heart failure or cardiac fibrosis. Of note, data obtained with ventricular myocytes from Gα_i2_-deficient mice indicate significant differences to mice lacking Gα_i3_ in signaling related to cardiac diseases, too (not shown). Proteomics analyses indicated increased phosphorylation of PLN in ventricular myocytes of β_1_-tg but not β_1_-tg/Gα_i3_^−/−^ mice. Our findings on PLN expression and phosphorylation suggest reduced SERCA inhibition in β_1_-tg hearts. This may be considered compensatory, as SERCA expression and activity are reduced in the setting of heart failure (del Monte and Hajjar 2008). In agreement with this, Engelhardt et al. found that genetic PLN ablation rescued β_1_-tg mice from heart failure (Engelhardt et al. 2004). It is tempting to speculate that the absence of compensatory PLN changes in β_1_-tg/Gα_i3_^−/−^ mice is indicative of cardioprotection by Gα_i3_ deficiency, as reduced PLN activity is not needed here. In Gα_i2_-deficient ventricles, our proteomics analysis revealed an increase of PLN phosphorylation similar to that in β_1_-tg specimens (not shown). This is interesting because in our previous study, Gα_i2_ deficiency alone already led to reduced life expectancy, but this effect was dramatically more pronounced when these animals also overexpressed the cardiac β_1_-AR (Keller et al. 2015).

Previously, we found a decreased density of ventricular L-type calcium currents (LTCC) in ventricular cardiomyocytes from Gα_i2_-deficient mice, while it was increased in Gα_i3_-deficient cardiomyocytes (Dizayee et al. 2011). Though in other models an increase of ventricular calcium currents led to cardiac damage and dysfunction in the long run (Muth et al. 1999; Nakayama et al. 2007; Beetz et al. 2009), Gα_i3_ deficiency does not impair cardiac function ((Jain et al. 2001) and this study). β_2_-Adrenoceptors couple to both G_s_ and G_i_ proteins, while G_s_ proteins are considered the cognate interaction partners of β_1_-adrenoceptors (Xiao et al. 1999; Seyedabadi et al. 2019). However, β_1_- and β_2_-adrenergic signaling seems to be modulated by G_i_ proteins including mechanisms independent of direct receptor coupling (Li et al. 2004; Martin et al. 2004; Melsom et al. 2014). Thus, it cannot be excluded that the above-mentioned differences between ventricular calcium currents in either Gα_i2_- or Gα_i3_-deficient mice also have a role in the development of cardiomyopathy in the β_1_-tg mouse model.

**Fig. 9:**
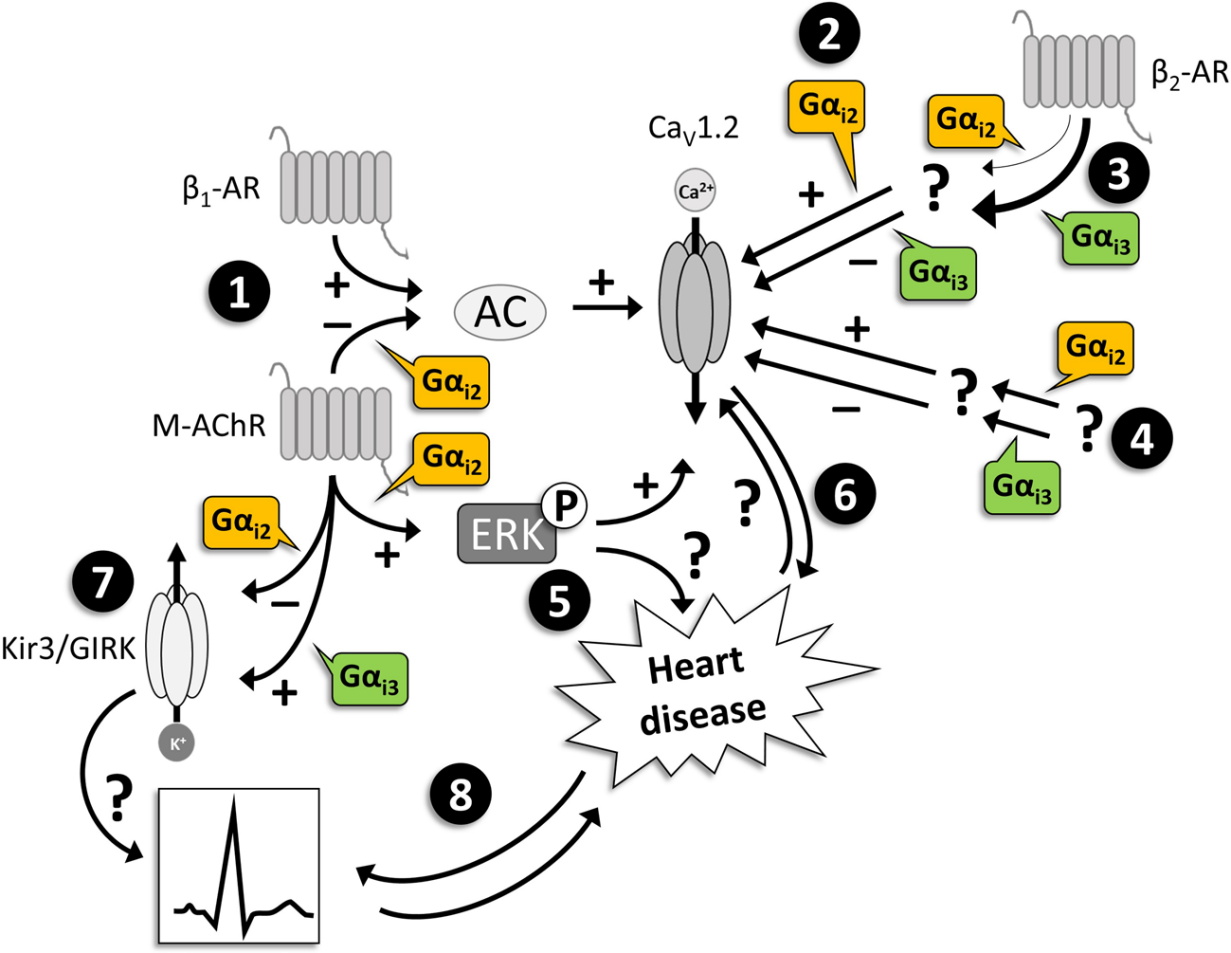
Isoform-specific Gα_i_ functions possibly involved in heart disease. 1: Gα_i2_, but not Gα_i3_, mediates signal transduction upon M-AChR stimulation and thereby may protect against β_1_-AR-mediated overstimulation, e.g., with respect to Ca^2+^ influx via L-type Ca^2+^ channels (Ca_V_1.2) (Nagata et al. 2000). 2: With increased signal transduction via β_2_-AR, Gα_i2_ increases the activity of individual Ca_V_1.2, while Gα_i3_ inhibits channel activity (Foerster et al. 2003; Klein 2009). 3: The coupling of β_2_-AR to Gα_i3_ may be stronger than to Gα_i2_, e.g., depending on the local membrane charge (Strohman et al. 2019). 4: Under basal conditions, Gα_i2_ appears to increase Ca_V_1.2-mediated I_CaL_ or to compensate for presumed inhibitory effects of Gα_i3_ and vice versa ((Dizayee et al. 2011), but: (Nagata et al. 2000)). 5: Gα_i2_, but not Gα_i3_, mediates phosphorylation of ERK and may thereby be involved in the stimulation of Ca_V_1.2 (Dizayee et al. 2011). The effect of ERK phosphorylation on heart disease has been described to be either harmful or protective, probably depending on the stimulus (Lorenz et al. 2009; Ruppert et al. 2013). Overall, G_i_ proteins differentially modulate Ca_V_1.2 and thus I_CaL_ via several isoform-specific mechanisms that depend among other things on the (level of) activity of β_1_-AR, β_2_-AR, and/or M-AChR. Alterations in Ca_V_1.2 activity and/or I_CaL_ have been associated with cardiomyopathy and heart failure (6). 7: Gα_i2_ deficiency led to an increase, Gα_i3_ deficiency to a decrease of Kir3/GIRK mediated currents (Nobles et al. 2018). Lack of Gα_i2_ thus might be pro-arrhythmic. Arrhythmia is a major reason of death in heart failure, and rhythm disturbances might cause or aggravate cardiomyopathy (8). “ + ” means stimulation/increase, “ − ” inhibition/decrease. “?” indicates that an interaction, a contribution or a consequence is not clear or fully understood. AC: adenylyl cyclase; β_1_-AR: β_1_-adrenoceptor; β_2_-AR: β_2_-adrenoceptor; ERK: extracellular signal-regulated kinase; Kir3/GIRK: inward-rectifier potassium channel/G protein-coupled inward-rectifier potassium channel; M-AChR: muscarinic acetylcholine receptor

The data discussed so far do not explain our findings regarding the opposing effects of Gα_i2_ and Gα_i3_, but PLN expression and activity as well as ventricular L-type calcium currents should be the subject of further investigations into possible molecular mechanisms underlying the differential effects of Gα_i_ isoforms in cardiomyopathy. Figure 9 and Table 3 summarize results on mechanisms that might contribute to isoform-specific signaling via inhibitory G-proteins in the heart.

**Table 3:** Possible isoform-specific effects of Gα_i2_ or Gα_i3_ in cardiac signaling observed in *Gnai*-deficient mice (italics: conclusions on the role of the respective Gα_i_ protein)

Gα_i_ isoform	Effector	Role of Gα_i_ deficiency	Role of Gα_ix_	Remark	Reference
Gα_i2_	I_CaL_	Blunted carbachol-mediated reversal of isoproterenol-induced I_CaL_ increase	*Mediates M-ACh-R signaling counteracting β-adrenergic I*_*CaL*_* stimulation*	Isolated ventricular myocytes from global Gα_i2_ and Gα_i3_ knockout mice	(Nagata et al. 2000)
Gα_i3_		Intact carbachol-mediated reversal of isoproterenol-induced I_CaL_ increase	*Not involved in M-ACh-R-mediated counter-regulation against β-adrenergic I*_*CaL*_* stimulation*		
Gα_i2_	I_CaL_	Decreased basal I_CaL_	*Increase of basal I*_*CaL*_* and/or counter-regulation of Gα*_*i3*_* effects*	Isolated ventricular myocytes from global Gα_i2_ and Gα_i3_ knockout mice	(Dizayee et al. 2011)
Gα_i3_		Increased basal I_CaL_	*Decrease of basal I*_*CaL*_* and/or counter-regulation of Gα*_*i2*_* effects*		
Gα_i2_	LTCC	Enhancement of the decrease in LTCC activity in β_2_-AR-overexpressing mice	*Stimulation of LTCC and/or counter-regulation of Gα*_*i3*_* effects*	Isolated ventricular myocytes; cardiac-specific β_2_-AR-overexpression; global, heterozygous Gα_i2_ knockout	(Foerster et al. 2003)
Gα_i3_	LTCC	Increased LTCC activity in β_2_-AR-overexpressing mice	*Inhibition of LTCC and/or counter-regulation of Gα*_*i2*_* effects*	Isolated ventricular myocytes; cardiac-specific β_2_-AR-overexpression; global, homozygous Gα_i3_ knockout	(Klein 2009)
Gα_i2_	Kir3/GIRK	Increased I_Kir_	*Inhibition of Kir3/GIRK channels and/or counter-regulation of Gα*_*i3*_* effects*	Isolated atrial myocytes; global Gα_ix_ knockouts	(Nobles et al. 2018)
Gα_i3_		Decreased I_Kir_	*Stimulation of Kir3/GIRK channels and/or counter-regulation of Gα*_*i2*_* effects*		
Gα_i2_	ERK	Blunted carbachol-induced phosphorylation of ERK	*Mediates M-AChR-induced ERK phosphorylation*	Ventricular homogenates; global Gα_ix_ knockouts; pre-treatment with carbachol in vivo	(Dizayee et al. 2011)
Gα_i3_		Unaffected carbachol-induced phosphorylation of ERK	*Not involved in M-AChR-medicated ERK phosphorylation*		

*β*_*2*_*-AR*, β_2_ adrenoceptor; *ERK*, extracellular signal-regulated kinase; *I*_*CaL*_, L-type Ca^2+^ currents; *I*_*Kir*_, Kir3/GIRK-mediated inward rectifying K^+^ current; *Kir3/GIRK*, inward-rectifier potassium channel/G protein-coupled inward-rectifier potassium channel; *LTCC*, L-type Ca^2+^ channel; *M-AChR*, muscarinic acetylcholine receptor

### Limitations of the study

The focus of our study centered on the hypothesis that Gα_i3_ and Gα_i2_ have different isoform-specific effects in a mouse model of dilated cardiomyopathy, despite sharing very high amino acid identity. In fact, we found significant functional differences between the two Gα_i_ isoforms. When evaluating these results, however, some methodological peculiarities must be considered that have an impact on the interpretation of the results.

Firstly, the data on qualitatively distinct differences in the effects of Gα_i2_ (Keller et al. 2015) and Gα_i3_ deficiency are based on two separate studies in which the gene-deficient mice were each tested against wildtype controls, but not directly against each other. Not least for animal welfare reasons, we were not able to retest a Gα_i2_-deficient cohort in our current study.

Furthermore, we used global Gα_i_ knockouts in the current and our previous study (Keller et al. 2015). Thus, we cannot exclude the possibility that extra-cardiac effects had an impact on the cardiac phenotype. However, previous studies comparing Gα_i2_- and Gα_i3_-deficient mice with their respective wildtype controls showed that, for example, basal heart rate or blood pressure was unchanged (Jain et al. 2001; Albarrán-Juárez et al. 2009). Furthermore, unaltered hypotensive effects following systemic α_2_-AR stimulation indicated normal circulatory regulation in Gα_i2_- and Gα_i3_-deficient mice, respectively (Albarrán-Juárez et al. 2009). We did not obtain catecholamine levels in our study. In a previous study, however, norepinephrine release from atria or brain cortex slices was not altered in Gα_i2_- or Gα_i3_-deficient mice (Albarrán-Juárez et al. 2009). Furthermore, given the unchanged basal values of heart rate and blood pressure in the absence of Gα_i2_ or Gα_i3_, significant changes in catecholamine levels seem rather unlikely (Jain et al. 2001; Albarrán-Juárez et al. 2009; Keller et al. 2015). Another study revealed the contribution of endogenous catecholamines to the phenotype of β_1_-tg mice to be negligible, thus arguing against a significant increase in catecholamine levels in this model, too (Engelhardt et al. 2001b).

The mouse model of β_1_-AR overexpression is a well-established and thoroughly characterized murine heart-failure model, but differs in some features from human heart failure. For example, there is up—instead of down—regulation of β_1_-AR (Bristow et al. 1986; Engelhardt et al. 1999). However, β_1_-AR overexpression can be considered as mimicking the chronically increased sympathetic stimulation observed in human heart failure (Engelhardt et al. 1999; Baker 2014). Although the transgenic approach displays a “non-physiologically” high β_1_-AR expression level, the (over-)expression levels in the C57BL/6-based mice we used here are significantly lower than on the FVB/N background on which the model was originally generated (Keller et al. 2015).

Here, we analyzed cardiac function only under basal conditions. A future study using stressors (e.g., dobutamine), could potentially reveal further differences between Gα_i_ isoforms, e.g., a possibly increased functional reserve in β_1_-tg mice lacking Gα_i3_.

Some of our results were supported by proteomics analysis (e.g., with respect to PLN, fibrosis, or hypertrophy). However, it should be noted that this was mainly a screening approach. Nevertheless, the results obtained may point to future studies on the role and molecular mechanisms of Gα_i2_- and Gα_i3_-mediated signaling in heart failure.

### Conclusion

Gα_i3_ deficiency has no detrimental effects in a mouse model of dilative cardiomyopathy and even appears to be cardio-protective. Our current and previous results indicate a β_1_-AR-mediated impairment whose development is oppositely associated with the expression of either Gα_i2_ or Gα_i3_. Although the underlying molecular mechanisms remain to be elucidated in further studies, our findings indicate isoform-specific interventions into G_i_-dependent signaling pathways (e.g., inhibiting Gα_i3_) to be promising novel strategies for cardio-protective therapies.

### Supplementary information

Below is the link to the electronic supplementary material.

Supplementary file1 (JPG 302 KB)

Supplementary file2 (JPG 416 KB)

Supplementary file3 (JPG 393 KB)

Supplementary file4 (JPG 514 KB)

Supplementary file5 (DOCX 13 KB)

Supplementary file6 (DOCX 13 KB)

Supplementary file7 (DOCX 16 KB)

## Data Availability

The datasets generated during and/or analyzed during the current study are available from the corresponding author on reasonable request.
