# Supplementary figures and images for: Protective effects of Gαi3 deficiency in a murine heart-failure model of β1-adrenoceptor overexpression
Source: Naunyn Schmiedebergs Arch Pharmacol. 2023 Oct 16;397(4):2401–20. doi: 10.1007/s00210-023-02751-8 (PMC10933181; doi:10.1007/s00210-023-02751-8)

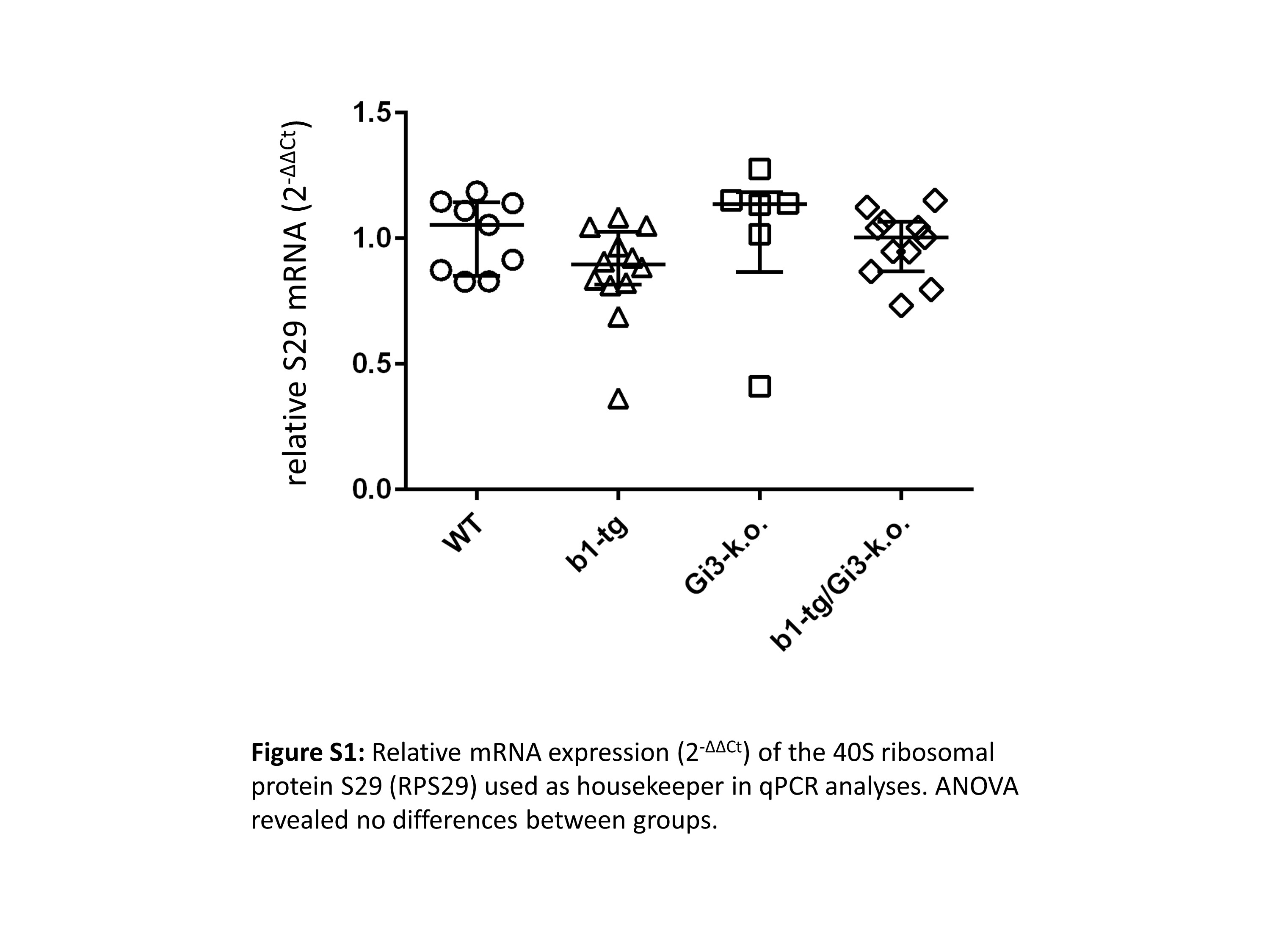

Supplement: Supplementary file 1 — Supplementary file1 (JPG 302 KB) [file 210_2023_2751_MOESM1_ESM.jpg]

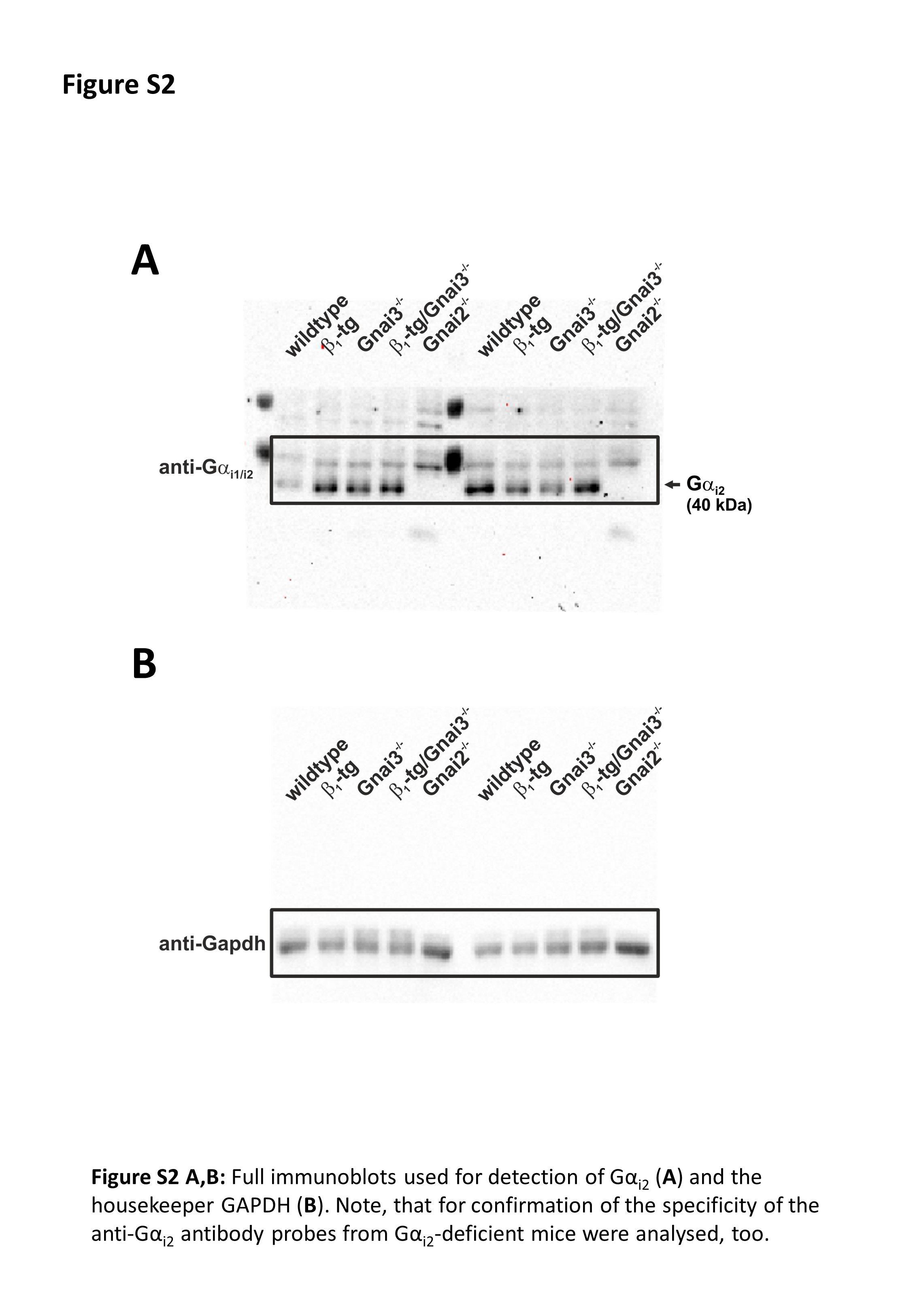

Supplement: Supplementary file 2 — Supplementary file2 (JPG 416 KB) [file 210_2023_2751_MOESM2_ESM.jpg]

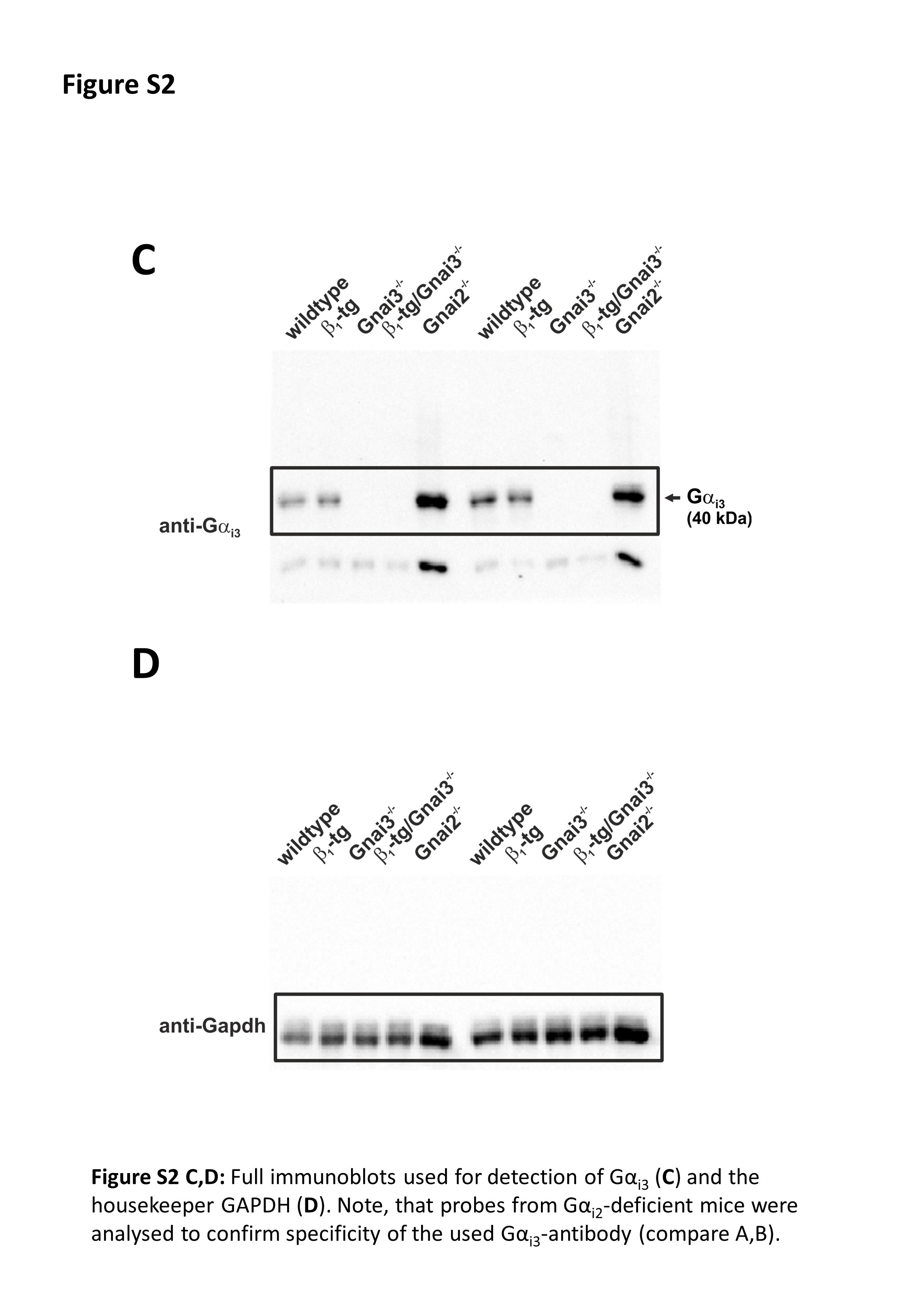

Supplement: Supplementary file 3 — Supplementary file3 (JPG 393 KB) [file 210_2023_2751_MOESM3_ESM.jpg]

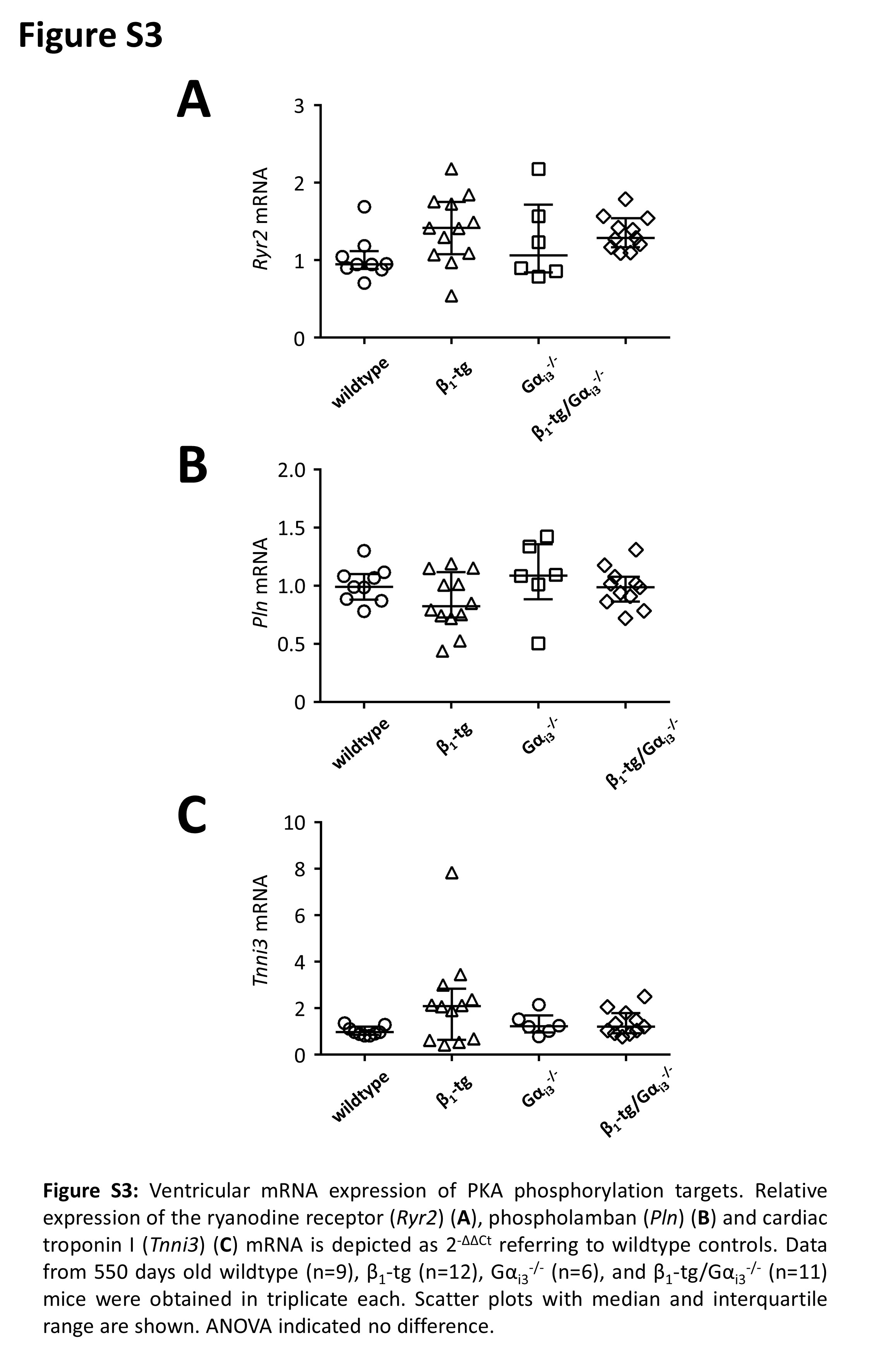

Supplement: Supplementary file 4 — Supplementary file4 (JPG 514 KB) [file 210_2023_2751_MOESM4_ESM.jpg]
