# Supplementary material for: Protective effects of Gαi3 deficiency in a murine heart-failure model of β1-adrenoceptor overexpression
Source: Naunyn Schmiedebergs Arch Pharmacol. 2023 Oct 16;397(4):2401–20. doi: 10.1007/s00210-023-02751-8 (PMC10933181; doi:10.1007/s00210-023-02751-8)
Supplement: Supplementary file 5 — Supplementary file5 (DOCX 13 KB) [file 210_2023_2751_MOESM5_ESM.docx]

| Parameter/investigation | wildtype | β_1_-tg | Gα_i3_^-/-^ | β_1_-tg/Gα_i3_^-/-^ |
| --- | --- | --- | --- | --- |
| ventricle- to body-weight ratio | 7/9 | 9/12 | 5/8 | 6/10 |
| fibrotic area | 3/0 | 3/0 | 2/1 | 2/2 |
| EF, LVESV, LVEDV, LVESL | 6/2 | 8/5 | 4/4 | 4/6 |
| GLS | 6/2 | 8/4 | 4/4 | 3/6 |
| E‘/A‘ | 6/2 | 7/4 | 4/4 | 4/6 |
| IVRT | 6/2 | 8/5 | 4/4 | 4/6 |
| qPCR | 3/6 | 5/7 | 3/3 | 4/7 |
| Western blot | 1/2 | 1/2 | 1/2 | 1/2 |
| proteomics | 3/0 | 3/0 | 3/0 | 3/0 |
| survival analysis: total | 193/215 | 124/138 | 82/72 | 37/45 |
| survival analysis: deaths | 9/8 | 24/28 | 7/4 | 8/5 |

**Table S1:** Sex distribution of mice used for the respective investigations (male/female).
