# Supplementary material for: Protective effects of Gαi3 deficiency in a murine heart-failure model of β1-adrenoceptor overexpression
Source: Naunyn Schmiedebergs Arch Pharmacol. 2023 Oct 16;397(4):2401–20. doi: 10.1007/s00210-023-02751-8 (PMC10933181; doi:10.1007/s00210-023-02751-8)
Supplement: Supplementary file 6 — Supplementary file6 (DOCX 13 KB) [file 210_2023_2751_MOESM6_ESM.docx]

| **Gene of interest** | **Sequence (5' → 3')** |
| --- | --- |
| *Nppa* (sense) | TCG TCT TGG CCT TTT GGC T |
| *Nppa* (antisense) | TCC AGG TGG TCT AGC AGG TTC T |
| *Nppb* (sense) | GTC AGT CGT TTG GGC TGT AAC |
| *Nppb* (antisense) | AGA CCC AGG CAG AGT CAG AA |
| *Gnai2* (sense) | AAG ACC TGT CCG GTG TCA T |
| *Gnai2* (antisense) | GGG ATG TAG TCA CTC TGT GC |
| *Gnai3* (sense) | TGG CTC TCA GTG ATT ACG ACC TT |
| *Gnai3* (antisense) | GGT TCA TTT CCT CAT CCT CAG C |
| *Pln* (sense) | CTG TGA CGA TCA CCG AAG C |
| *Pln* (antisense) | TGG TCA AGA GAA AGA TAA AAA GTT GA |
| *Ryr2* (sense) | TTC ACA CCT GTT CCT GTG GA |
| *Ryr2* (antisense) | TTT CTC TTA TCC TTT CCA GGT GA |
| *Rps29* (forward) | ATG GGT CAC CAG CAG CTC TA |
| *Rps29* (reverse) | AGC CTA TGT CCT TCG CGT ACT |
| *Tnni3* (sense) | GAG CCA CAC GCC AAG AAA |
| *Tnni3* (antisense) | GCC CCT TCT CTC CAC GTC |

**Table S2:** Primer pairs used for qPCR.
