# Supplementary material for: Protective effects of Gαi3 deficiency in a murine heart-failure model of β1-adrenoceptor overexpression
Source: Naunyn Schmiedebergs Arch Pharmacol. 2023 Oct 16;397(4):2401–20. doi: 10.1007/s00210-023-02751-8 (PMC10933181; doi:10.1007/s00210-023-02751-8)
Supplement: Supplementary file 7 — Supplementary file7 (DOCX 16 KB) [file 210_2023_2751_MOESM7_ESM.docx]

|  | Wildtype  vs. β_1_-tg | Wildtype  vs. Gα_i3_^-/-^ | Wildtype  vs. β_1_-tg/Gα_i3_^-/-^ | β_1_-tg  vs. β_1_-tg/Gα_i3_^-/-^ |
| --- | --- | --- | --- | --- |
| ventricle- to body-weight ratio | **0.773***  **(0.099 – 1.447)** | **0.915**  **(0.132 – 1.58)** | **0.586**  **(0.132 – 1.58)** | -0.38  (-1.037 – 0.276) |
| ejection fraction | **-1.642***  **(-2.653 – -0.631)** | 0.304  (-0.681 – 1.29) | -0.585  (-1.534 – 0.365) | **0.962***  **(0.092 – 1.832)** |
| LVESV | **0.985**  **(0.055 – 1.915)** | -0.467  (-1.46 – 0.526) | 0.446  (-0.495 – 1.387) | -0.647  (-1.492 – 0.198) |
| LVEDV | 0.702  (-0.204 – 1.608) | -0.508  (-1.503 – 0.488) | 0.197  (-0.735 – 1.129) | -0.523  (-1.361 – 0.315) |
| LVESL | **1.413***  **(0.434 – 2.392)** | 0.305  (-0.681 – 1.29) | 0.904  (-0.071 – 1.88) | -0.511  (-1.348 – 0.327) |
| GLS | **-1.409***  **(-2.404 – -0.413)** | -0.017  (-0.997 – 0.963) | -0.43  (-1.393 – 0.533) | 0.801  (-0.097 – 1.699) |
| E’/A’ | **-1.057**  **(-2.027 – -0.086)** | -0.659  (-1.665 – 0.348) | 0.147  (-0.784 – 1.078) | **1.352***  **(0.403 – 2.301)** |
| IVRT | **1.633***  **(0.623 – 2.643)** | -0.418  (-1.409 – 0.572) | -0.456  (-1.398 – 0.486) | **-1.805***  **(-2.781 – -0.83)** |
| fibrotic area | **9.377***  **(3.836 – 14.919)** | **2.253**  **(0.207 – 4.3)** | **5.59***  **(2.301 – 8.879)** | **-3.063***  **(-5.257 – -0.868)** |
| *Nppa* mRNA log10 | **3.605^#^**  **(2.214 – 4.997)** | **1.346**  **(0.206 – 2.485)** | **2.208^#^**  **(1.092 – 3.323)** | **-1.368^#^**  **(-2.276 – -0.459)** |
| *Nppb* mRNA log10 | **1.513^#^**  **(0.535 – 2.491)** | 0.74  (-0.236 – 1.807) | **1.883^#^**  **(0.826 – 2.94)** | -0.384  (-1.209 – 0.442) |
| Gnai2 mRNA | **1.46^#^**  **(0.489 – 2.43)** | **1.148**  **(0.036 – 2.259)** | **1.633**  **(0.617 – 2.649)** | **-0.914^#^**  **(-1.774 – -0.054)** |
| *Gnai3* mRNA | **1.452^§^**  **(0.482 – 2.421)** | --- | --- | --- |
| *Ryr2* mRNA | **0.973**  **(0.06 – 1.886)** | 0.563  (-0.49 – 1.615) | **1.301**  **(0.332 – 2.27)** | -0.146  (-0.965 – 0.673) |
| *Pln* mRNA | -0.692  (-1.581 – 0.197) | 0.282  (-0.756 – 1.32) | -0.163  (-1.046 – 0.719) | 0.565  (-0.269 – 1.4) |
| *Tnni3* mRNA | 0.813  (-0.085 – 1.712) | 0.92  (-0.164 – 2.004) | 0.822  (-0.095 – 1.739) | -0.599  (-1.435 – 0.238) |
| Gα_i2_ protein | -0.373  (-1.987 – 1.241) | -0.362  (-1.975 – 1.252) | -0.034  (-1.635 – 1.566) | 0.292  (-1.317 – 1.901) |
| Gα_i3_ protein | -0.247  (-1.36 – 1.853) | --- | --- | --- |
| PLN protein | **-2.927**  **(-5.229 – -0.624)** | **-2.002**  **(-3.963 – -0.041)** | 0.184  (-1.42 – 1.787) | **2.221**  **(0.186 – 4.256)** |
| pAkt/Akt ratio | -1.431  (-3.225 – 0.362) | -1.137  (-2.862 – 0.588) | -0.64  (-2.28 – 1.001) | (1.211  (-0.53 – 2.952) |

**Table S3:** Effects sizes calculated as Cohen’s d are shown with respective 95% confidence intervals. Estimations are based upon difference of means of two groups of different or equal size as appropriate. **Bold**: 95%CI does not include zero. Symbols indicate statistically significant differences obtained in Bonferroni (*) or Holm-Šídák (^#^) post-tests following ANOVA or in a Mann-Whitney test (^§^) applied on underlying raw data, respectively. Data were obtained from animals at an age of about 550 days.
